# Mitral repair versus replacement: 20-year outcome trends in the UK (2000–2019)

**DOI:** 10.1093/icvts/ivad086

**Published:** 2023-05-19

**Authors:** Fadi Al-Zubaidi, Maria Pufulete, Shubhra Sinha, Simon Kendall, Narain Moorjani, Massimo Caputo, Gianni D Angelini, Hunaid A Vohra

**Affiliations:** Department of Cardiac Surgery, Bristol Heart Institute, Bristol, UK; Faculty of Health Sciences, University of Bristol, Bristol Heart Institute, Bristol, UK; Department of Cardiac Surgery, Bristol Heart Institute, Bristol, UK; Department of Cardiac Surgery, South Tees Hospital, Newcastle, UK; Department of Cardiac Surgery, Royal Papworth Hospital, Cambridge, UK; Department of Cardiac Surgery, Bristol Heart Institute, Bristol, UK; Department of Cardiac Surgery, Bristol Heart Institute, Bristol, UK; Department of Cardiac Surgery, Bristol Heart Institute, Bristol, UK

**Keywords:** Mitral, Repair, Replacement, Mortality, Trends, UK

## Abstract

**OBJECTIVES:**

Using a large national database, we sought to describe outcome trends in mitral valve surgery between 2000 and 2019.

**METHODS:**

The study cohort was split into mitral valve repair (MVr) or replacement, including all patients regardless of concomitant procedures. Patients were grouped by four-year admission periods into groups (A to E). The primary outcome was in hospital mortality and secondary outcomes were return to theatre, postoperative stroke and postoperative length of stay. We investigated trends over time in patient demographics, comorbidities, intraoperative characteristics and postoperative outcomes. We used a multivariable binary logistic regression model to assess the relationship between mortality and time. Cohorts were further stratified by sex and aetiology.

**RESULTS:**

Of the 63 000 patients in the study cohort, 31 644 had an MVr and 31 356 had a replacement. Significant demographic shifts were observed. Aetiology has shifted towards degenerative disease; endocarditis rates in MVr dropped initially but are now rising (period A = 6%, period C = 4%, period E = 6%; *P* < 0.001). The burden of comorbidities has increased over time. In the latest time period, women had lower repair rates (49% vs 67%, *P* < 0.001) and higher mortality rates when undergoing repair (3% vs 2%, *P* = 0.001) than men. Unadjusted postoperative mortality dropped in MVr (5% vs 2%, *P* < 0.001) and replacement (9% vs 7%, *P* = 0.015). Secondary outcomes have improved. Time period was an independent predictor for reduced mortality in both repair (odds ratio: 0.41, 95% confidence interval: 0.28–0.61, *P* < 0.001) and replacement (odds ratio: 0.50, 95% confidence interval: 0.41–0.61, *P* < 0.001).

**CONCLUSIONS:**

In-hospital mortality has dropped significantly over time for mitral valve surgery in the UK. MVr has become the more common procedure. Sex-based discrepancies in repair rates and mortality require further investigation. Endocarditis rates in MVS are rising.

**Figure:**
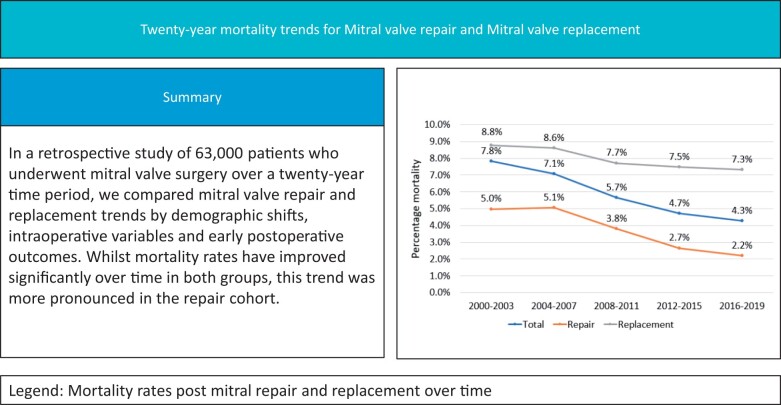

## INTRODUCTION

Over the past two decades, mitral valve surgery (MVS) in the UK has evolved as a sub-specialty of cardiothoracic surgery in its own right. Patients requiring MVS are increasingly older and more likely to present with multiple co-morbidities [1]. Over time aetiologies of MV disease have shifted, with degenerative disease now making up the majority of cases requiring elective MVS. There is now general agreement that mitral valve repair (MVr) is superior to mitral valve replacement (MVR) for degenerative disease [2–9].

The Society for Cardiothoracic Surgery published UK trends in MVS between 2002 and 2019 in their January 2021 National Cardiac Surgery Activity and Outcomes Report (NACSA) [10] in which overall trends in mortality, repair rates and patient demographics were reported. This article seeks to expand on this report, focusing on differences between MVr and MVR. We aimed to stratify the MVS population by procedure type and analyse how patient demographics, intraoperative variables and postoperative outcomes have changed for MVr and MVR patients in the UK between 2000 and 2019. Finally, we sought to describe trends by aetiology of mitral disease and by patient sex.

## MATERIALS AND METHODS

### Ethics statement

The register-based cohort study is part of a research approved by the Health Research Authority and Health and Care Research Wales and a need for patients’ consent was waived, as all patients in the database were anonymized (HCRW) (IRAS ID: 257758, 23 July 2019).

### Data sources

Our analyses were conducted on NACSA data, maintained by the National Institute for Cardiovascular Outcomes Research (NICOR) [10]. This national database contains clinical information on demographics, pre- and postoperative clinical information including mortality, for all major adult cardiac surgery procedures performed in the UK [10]. Maintenance and validation are regularly undertaken by the use of reproducible cleaning and maintenance algorithms, with return to individual centres for local validation [11]. We obtained approval to conduct this study from NICOR and from our institution, the University Hospitals Bristol and Weston National Health Service Foundation Trust Clinical Audit Team, to carry out this research without requiring patient informed consent. The data were received with all identifiable patient information removed; the requirement for patient informed consent was waived and the dataset was adapted for use with statistical analysis software.

### Study population

Patients undergoing MVS between 2000 and 2019, including concomitant TV surgery, coronary artery bypass grafting and ablation for atrial fibrillation (AF).

### Outcomes

In-hospital mortality was the primary outcome. Secondary outcomes were rates of return to theatre, postoperative stroke and length of hospital stay.

### Data cleaning and dealing with missing data

The study population dataset was resurveyed for non-adult or duplicate data and removed as appropriate. Ambiguously coded, conflicting or extreme outlying values were recoded as missing data. The overall percentage of missing data for baseline information was <5%. Little’s test was performed and data were found not to be missing completely at random; given the nature of the data collection, missingness was deemed likely to be related to the true value i.e. ‘did not occur’. We therefore re-coded these missing datapoints as ‘0’.

### Statistical analysis

Analyses were performed using IBM SPSS Statistics for Windows, version 26 (IBM Corp., Armonk, NY, USA). The overall MVS population was initially stratified into two groups according to procedure type: MVr and MVR. We split the MVr and MVR populations into five 4-year time periods: A (2000–2003), B (2004–2007), C (2008–2011), D (2012–2015) and E (2016–2019) according to the date of procedure. Descriptive statistics were used to summarize the characteristics of the MVr and MVR populations; categorical variables were summarized as counts and percentages; normally distributed continuous variables were summarized as means and standard errors and non-normally distributed variables were summarized as median and the interquartile range. For the time-trends analysis, we analysed unadjusted trends for all variables and outcome measures. Trends in categorical variables and continuous variables between the time periods were investigated Cochran–Mantel–Haenszel tests or simple linear regression respectively. Significance was defined as *P* < 0.05.

The overall MVS group was split by sex and by aetiology of disease; descriptive statistics were performed for these selected variables. We sought to separately describe trends in repair rates, operative time and mortality by sex and aetiology of disease.

We then planned binary logistic regression models with mortality as the dependent variable in the original MVr and MVR groups to investigate for an independent relationship between time and mortality. Within each cohort, univariable trends were first assessed as described as stated above; variables associated with 4-year admission period (defined as *P* < 0.25) were then included with the variable ‘4-year admission period’ as covariates in the binary regression models. Period A was selected as the reference group. Adjusted relationships between 4-year admission period and mortality were expressed as odds ratios (OR) with 95% confidence intervals (CIs).

## RESULTS

Of the 63 809 patients undergoing MVS between 2000 and 2019, 809 had missing admission dates, leaving 63 000 patients in the analysis, of whom 31 644 (50%) underwent MVr and 31 356 (50%) underwent MVR.

### Overall trends in mitral surgery

**Figure 1:**
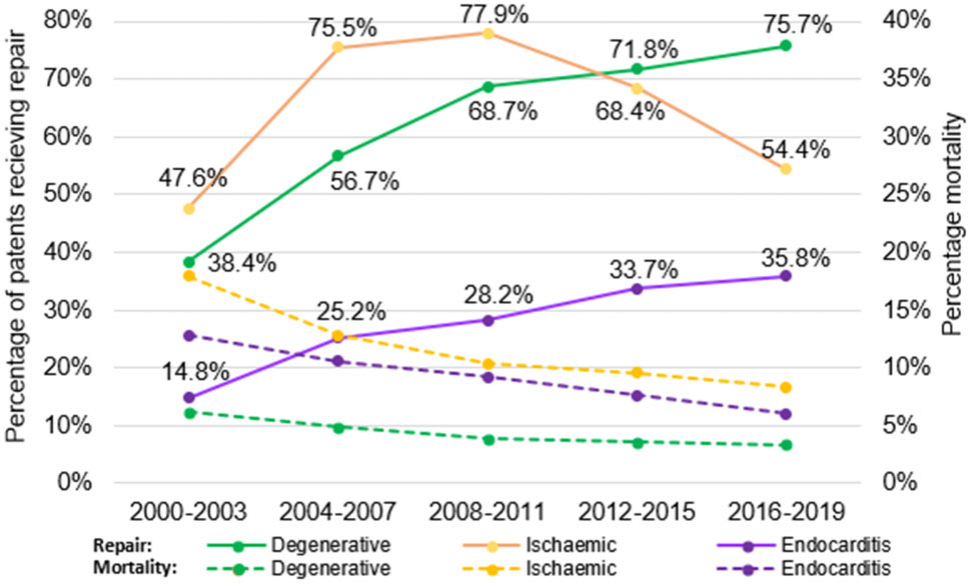
Mitral repair and mortality rates by aetiology of disease.

Baseline patient demographics, comorbidities and intraoperative characteristics over time are shown in Tables 1 and 2 (see supplementary material for overall mitral population data). There was a significant shift in demographics for both MVr and MVR from period A to period E (Table 1); patients presenting for surgery were on average older with a higher BMI and the proportion of octogenarians increased from 4% to 11% for MVr and 3% to 11% for MVR (*P* < 0.001). Patients were less likely to present with EF range <50% in both MVr (45–27%, *P* < 0.001) and MVR (36–34%, *P* < 0.001). Degenerative disease as the indication for mitral surgery increased over time; from 53% to 67% (*P* < 0.001) in the MVr group and 25% to 31% (*P* < 0.001) in MVR. In MVr patients, endocarditis dropped from 6% to 4% between periods A and C, before rising again to 6% by period E (*P* < 0.001); in MVR, the indication of endocarditis rose steadily from 10% to 15% (*P* < 0.001). Of the patients who had MVS for endocarditis, 13% had previous MV surgery: period A: 16%; period B: 18%, period C: 16%, period D: 12%; period E: 9%. Rates of surgery for ischaemic and rheumatic disease both dropped over time. Rates of mitral stenosis have dropped in both MVr (4–2%, *P* < 0.001) and MVR (42–38%, *P* < 0.001) groups. Accordingly, repair rates for degenerative disease have risen sharply over the observed period, from 38% in period A to 72% in period D (see Fig. 1).

**Table 1:** Trends in baseline demographics, cardiovascular and heart failure scores and mitral valve pathology of patients undergoing mitral valve repair and mitral valve replacement

Period	A: 2000–2003 (7200)		B: 2004–2007 (12 276)		C: 2008–2011 (14 521)		D: 2012–2015 (14 945)		E: 2016–2019 (14 058)		Trends *P*-value (all years)	
Variable	MVr (*n* = 1779)	MVR (*n* = 5421)	MVr (*n* = 5349)	MVR (*n* = 6927)	MVr (*n* = 7617)	MVR (*n* = 6904)	MVr (*n* = 8555)	MVR (*n* = 6390)	MVr (*n* = 8344)	MVR (*n* = 5714)	MVr (*n* = 31 644)	MVR (*n* = 31 356)
Age (years)^a^	65.6 (±11.4)	64.3 (±11.6)	66.1 (±12.0)	65.1 (±12.3)	67.0 (±12.1)	65.6 (±13.0)	67.0 (±12.3)	65.5 (±13.5)	67.3 (±12.1)	65.3 (±13.4)	<0.001^b^	<0.001^b^
BMI (kg/m^2^)^a^	25.7 (±4.6)	25.3 (±4.5)	26.0 (±4.4)	26.0 (±5.2)	26.3 (±4.5)	26.5 (±5.2)	26.5 (±4.8)	26.9 (±5.3)	26.4 (±4.8)	27.2 (±5.9)	<0.001	<0.001
Female sex, *n* (%)	594 (33)	3004 (55)	1801 (34)	3885 (56)	2660 (35)	3790 (55)	2845 (33)	3463 (54)	2759 (33)	2903 (51)	0.208	<0.001
Octogenarian, *n* (%)	73 (4)	145 (3)	363 (7)	337 (5)	743 (10)	532 (8)	919 (11)	674 (11)	940 (11)	614 (11)	<0.001	<0.001
Active smoker, *n* (%)	103 (6)	463 (9)	314 (6)	635 (9)	452 (6)	648 (10)	487 (6)	665 (11)	465 (6)	670 (12)	0.268	<0.001
EF range, *n* (%)											<0.001	<0.001
<50%	780 (45)	1871 (36)	2292 (44)	2564 (38)	2856 (38)	2455 (36)	2755 (33)	2258 (36)	2245 (27)	1930 (34)		
NYHA score, *n* (%)											<0.001	0.661
I–II	884 (51)	1832 (34)	2839 (54)	2326 (34)	3959 (52)	2488 (36)	4258 (50)	2259 (36)	4621 (52)	1963 (34)		
III–IV	864 (50)	3486 (66)	2426 (46)	4451 (66)	3625 (48)	4366 (64)	4276 (50)	4100 (65)	4022 (48)	3751 (66)		
CCS score, *n* (%)											<0.001	<0.001
0	787 (56)	2842 (57)	2731 (60)	4054 (64)	4829 (64)	4424 (65)	5934 (70)	4263 (67%)	6063 (73)	3864 (68)		
I–II	394 (28)	1526 (31)	1232 (27)	1698 (27)	1985 (26)	1838 (27)	1902 (22)	1562 (25)	1753 (21)	1349 (25)		
III–IV	215 (15)	584 (12)	608 (13)	624 (10)	733 (10)	570 (8)	700 (8)	540 (9)	528 (6)	501 (9)		
MV stenosis	68 (4)	2091 (42)	107 (2)	2768 (42)	201 (3)	2787 (41)	121 (2)	2318 (38)	101 (1)	1833 (33)	0.009	0.170
MV pathology, *n* (%)												
Degenerative	660 (53)	1058 (25)	2596 (57)	1980 (33)	4525 (65)	2057 (34)	5008 (64)	1967 (34)	5299 (67)	1699 (31)	<0.001	<0.001
Rheumatic	75 (6)	1808 (42)	150 (3)	2345 (40)	189 (3)	2286 (38)	181 (2)	2080 (36)	117 (1)	1783 (33)	<0.001	<0.001
Ischaemic	202 (16)	222 (5)	670 (15)	218 (4)	753 (11)	214 (4)	605 (8)	280 (5)	454 (6)	381 (7)	<0.001	<0.001
Functional	41 (3)	52 (1)	464 (10)	103 (2)	732 (11)	159 (3)	849 (11%)	155 (3) )	869 (11)	256 (5)	<0.001	<0.001
Endocarditis	70 (6)	404 (10)	198 (4)	587 (10)	270 (4)	686 (11)	365 (5)	716 (12)	468 (6)	838 (15)	<0.001	<0.001
Other	178 (14)	640 (15)	363 (8)	643 (11)	312 (5)	556 (9)	709 (9)	504 (8)	719 (9)	518 (10)	0.458	<0.001

Missingness: age = 11 (0%); BMI = 2122 (3%); sex = 6 (0%); octogenarian = 11 (0%); active smoker = 744 (1%); EF range = 995 (2%); NYHA score = 502 (1%); CCS score = 2366 (4%); MV stenosis = 2640 (4%); MV pathology = 7008 (11%).

BMI: body mass index; CCS: Canadian Cardiovascular Society; EF: ejection fraction; MV: mitral valve; MVr: mitral valve repair; MVR: mitral valve replacement; NYHA: New York Heart Association.

a Continuous variables expressed as mean ± standard deviation.

b Cochran–Mantel–Haenszel and linear regression for trends, comparing the time periods.

**Table 2:** Trends in baseline comorbidities and intraoperative characteristics of patients undergoing mitral valve repair and mitral valve replacement

Period	A: 2000–2003 (7200)		B: 2004–2007 (12 276)		C: 2008–2011 (14 521)		D: 2012–2015 (14 945)		E: 2016–2019 (14 058)		Trends *P*-value (all years)	
Variable	MVr (*n* = 1779)	MVR (*n* = 5421)	MVr (*n* = 5349)	MVR (*n* = 6927)	MVr (*n* = 7617)	MVR (*n* = 6904)	MVr (*n* = 8555)	MVR (*n* = 6390)	MVr (*n* = 8344)	MVR (*n* = 5714)	MVr (*n* = 31 644)	MVR (*n* = 31 356)
Diabetes	139 (8)	514 (10)	533 (10)	812 (12)	747 (10)	914 (13)	897 (11)	946 (15) )	715 (9)	850 (15)	0.358	<0.001
Coronary disease	561 (41)	1499 (33)	1806 (42)	1792 (32)	2376 (37)	1737 (23)	2341 (31)	1575 (28)	1790 (25)	1278 (26)	<0.001	<0.001
Hypertension	737 (42)	1786 (34)	2613 (50)	2934 (43)	4048 (54)	3386 (50)	4703 (55)	3389 (54)	4461 (53)	3175 (56)	<0.001	<0.001
COPD	188 (11)	797 (15)	601 (11)	1193 (18)	954 (13)	1286 (19)	1114 (13)	1115 (18)	869 (11)	929 (17)	0.750	0.025
Previous stroke/TIA	93 (7)	423 (9)	293 (6)	682 (11)	488 (7)	781 (12)	649 (8)	758 (12)	572 (7)	706 (13)	0.145	<0.001
Previous MI	260 (15)	678 (13)	1036 (20)	759 (11)	1255 (17)	771 (11)	1131 (13)	194 (13)	814 (10)	711 (12)	<0.001	0.967
Redo MV surgery	41 (2)	513 (10)	304 (6)	900 (13)	562 (7)	994 (14)	902 (11)	867 (14)	738 (9)	525 (9)	0.040	0.933
PVD	96 (6)	293 (6)	361 (7)	439 (6)	486 (6)	456 (7)	430 (5)	426 (7)	317 (4)	341 (6)	<0.001	0.413
AF/flutter	562 (32)	2536 (48)	1647 (31)	3196 (48)	2336 (33)	2979 (46)	2719 (34)	2460 (41)	2666 (32)	2091 (37)	0.671	<0.001
CPB time,^a^ mean (±SD)	120.4 (±1.2)	123.9 (±0.8)	132.4 (±0.8)	136.3 (±0.8)	133.3 (±0.6)	145.7 (±0.9)	132.8 (±0.6)	152.6 (±0.9)	130.8 (±0.6)	150.1 (±1.0)	<0.001	<0.001
Cross-clamp time,^a^ mean (±SD)	86.7 (±0.8)	87.7 (±0.6)	96.7 (±0.6)	98.4 (±0.6)	99.1 (±0.5)	106.6 (±0.6)	99.7 (±0.5)	112.4 (±0.7)	96.1 (±0.4)	112.4 (±0.7)	<0.001	<0.001
Urgency											<0.001	<0.001
Elective	1366 (77)	3851 (72)	4159 (78)	4987 (72)	6068 (80)	5051 (73)	6812 (80)	4304 (67)	6664 (80)	3765 (66)		
Urgent	356 (20)	1194 (22)	1074 (20)	1500 (22)	1417 (19)	1493 (22)	1632 (19)	1740 (27)	1564 (19)	1616 (28)		
Emergency	55 (3)	336 (6)	105 (2)	370 (5)	125 (2)	316 (5)	96 (1)	290 (5)	101 (1)	293 (5)		
Salvage	1 (0)	31 (1)	10 (0)	56 (1)	9 (0)	43 (1)	14 (0)	53 (1)	12 (0)	39 (1)		
Preop shock	24 (2)	186 (4)	75 (2)	238 (4)	89 (1)	221 (3)	87 (1)	227 (4)	81 (1)	222 (4)	<0.001	0.942
Preop dialysis	0 (0)	0 (0)	0 (0)	1 (5)	18 (1)	46 (3)	85 (1)	147 (3)	73 (1)	164 (3)	<0.001	<0.001
Concom. ablation^b^	1 (0)	0 (0)	0 (0)	0 (0)	197 (8)	87 (3)	879 (32)	334 (14)	831 (31)	247 (12)	<0.001	<0.001
Concom. CABG	616 (35)	1381 (26)	1883 (35)	1682 (24)	2706 (36)	1765 (26)	2270 (27)	1400 (22)	1766 (21)	1156 (20)	<0.001	<0.001
Concom. TV surg	118 (7)	353 (7)	696 (13)	940 (14)	1377 (18)	1227 (18)	1751 (21)	1280 (20)	1737 (21)	1146 (20)	<0.001	<0.001

Numbers are *n* (%). Missingness: diabetes = 370 (1%); coronary disease = 9543 (15%); hypertension = 587 (1%); COPD = 810 (1%); previous stroke/TIA = 3258 (5%); previous MI = 1087 (2%); redo sternotomy = 3332 (5%); PVD = 483 (1%); AF/flutter = 2253 (4%); cross-clamp time = 2248 (4%); CPB time = 2246 (4%); urgency = 31 (0%); concom. ablation = 14 407 (23%); concom. CABG = 16 059 (49%); concom. TV surg = 40 872 (65%).

a Continuous variables are expressed as mean ± standard error of mean.

b Ablation rates reported for patients with preoperative AF.

AF: atrial fibrillation; CABG: coronary artery bypass graft; COPD: chronic obstructive pulmonary disease; CPB: cardiopulmonary bypass; MI: myocardial infarction; MV: mitral valve; MVr: mitral valve repair; MVR: mitral valve replacement; PVD: peripheral vascular disease; TIA: transient ischaemic attack; TV: tricuspid valve; SD: standard deviation.

For both MVr and MVR, the proportion of patient with comorbidities increased over time (Table 2). In terms of preoperative AF/flutter, there was no change over time in patients receiving MVr (32–32%, *P* = 0.671); however, in patients receiving MVR, the proportion with AF/flutter decreased from 48% to 37% (*P* < 0.001). Rates of concomitant ablation for patients with preoperative AF rose significantly in both groups, from 0% to 31% in MVr (*P* < 0.001) and 0% to 12% in MVR (*P* < 0.001).

Trends in urgency of admission changed significantly over time (Table 2); MVr cases were more likely to be elective over time, increasing from 77% to 80%, *P* < 0.001. Urgent MVr dropped slightly from 20% to 19%, *P* < 0.001. MVR cases conversely were less likely to be elective, with elective MVR dropping from 72% to 66%, *P* < 0.001. Urgent MVR increased from 22% to 28%, *P* < 0.001.

Rates of TV surgery increased significantly in both groups, from 7% to 21%, *P* < 0.001, in MVr and 7% to 20%, *P* < 0.001, in MVR. Conversely, rates of concomitant coronary artery bypass grafting decreased in both groups, from 35% to 21% (*P* < 0.001) in MVr and 26% to 20% (*P* < 0.001) in MVR. Cardiopulmonary bypass (CPB) times and aortic cross-clamp (AoX) times increased over time in both cohorts (*P* < 0.001); MVR had consistently longer CPB and AoX times than MVr.

### Trends in postoperative outcomes

There were significant changes in intraoperative and postoperative outcomes over time (see Tables 2 and 3). Unadjusted post-operative mortality decreased over time in both MVr (5% vs 2%, *P* < 0.001) and MVR (9% vs 7%, *P* = 0.015). Rates of postoperative return to the operating theatre decreased in both MVr (8% vs 6%, *P* = 0.001) and MVR (10% vs 7%, *P* < 0.001). Postoperative stroke remained low over time, remaining at 1% in MVr and 2% in MVR. MVr patients were less likely to require postoperative dialysis over time (5% vs 3%, *P* < 0.001); however, there was no significant trend in the MVR group. Length of hospital stay dropped in the MVr cohort (10 vs 7 days, *P* < 0.001); however, there was no significant change in MVR (10 vs 10 days, *P* = 0.254).

**Table 3:** Trends in postoperative characteristics of patients undergoing mitral valve repair and mitral valve replacement

Period	A: 2000–2003 (7200)		B: 2004–2007 (12 276)		C: 2008–2011 (14 521)		D: 2012–2015 (14 945)		E: 2016–2019 (14 058)		Trends *P*-value (all years)		
Variable	MVr (*n* = 1779)	MVR (*n* = 5421)	MVr (*n* = 5349)	MVR (*n* = 6927)	MVr (*n* = 7617)	MVR (*n* = 6904)	MVr (*n* = 8555)	MVR (*n* = 6390)	MVr (*n* = 8344)	MVR (*n* = 5714)	MVr (*n* = 31 644)	MVR (*n* = 31 356)	
Mortality, *n* (%)	88 (5)	471 (9)	270 (5)	596 (8.6)	288 (4)	525 (8)	221 (3)	467 (8)	181 (2)	410 (7)	<0.001	0.015	
Takeback, *n* (%)	119 (8)	444 (10)	393 (8)	617 (10)	497 (7)	641 (10)	512 (6)	584 (10)	379 (5)	415 (7)	<0.001	<0.001	
Stroke, *n* (%)	17 (1)	103 (2)	39 (1)	96 (2)	61 (1)	114 (2)	64 (1)	88 (2)	63 (1)	106 (2)	<0.001	<0.001	
Postop dialysis, *n* (%)	61 (5)	303 (7)	265 (6)	470 (8)	325 (5)	507 (9)	301 (4)	448 (8)	229 (3)	418 (8)	<0.001	0.580	
Length of admission (days), median (IQR)	10 (7–15)	10 (7–15)	10 (7–15)	10 (7–16)	9 (6–14)	9 (6–14)	9 (7–15)	9 (7–15)	7 (5–11)	10 (7–17)	<0.001	0.254	

Missingness: mortality = 855 (1%); takeback = 4713 (8%); stroke = 9012 (14%); postop dialysis = 8186 (13%); length of admission = 413 (1%).

IQR: interquartile range; MVr: mitral valve repair; MVR: mitral valve replacement.

### Adjusted postoperative mortality

After adjusting for confounders, by period E, there was a statistically significant reduction in postoperative mortality in both MVr (OR: 0.41, 95% CI: 0.28–0.61, *P* < 0.001) and MVR cohorts (OR: 0.50, 95% CI: 0.41–0.61, *P* < 0.001) (see Table 4).

**Table 4:** Odds ratios and 95% confidence intervals for mortality over time in mitral valve repair and replacement groups

Cohort	Odds ratio ± 95% CI	*P*-Value
Mitral valve surgery (total cohort)		
2000–2003 (reference)		
2004–2007	0.75 (0.63–0.88)	<0.001
2008–2011	0.59 (0.50–0.70)	<0.001
2012–2015	0.48 (0.41–0.57)	<0.001
2016–2019	0.44 (0.32–0.66)	<0.001
Mitral valve repair		
2000–2003 (reference)		
2004–2007	0.80 (0.55–1.16)	0.358
2008–2011	0.62 (0.43–0.90)	<0.001
2012–2015	0.49 (0.33–0.73)	<0.001
2016–2019	0.41 (0.28–0.61)	<0.001
Mitral valve replacement		
2000–2003 (reference)		
2004–2007	0.78 (0.64–0.95)	0.035
2008–2011	0.65 (0.54–0.80)	<0.001
2012–2015	0.54 (0.43–0.67)	<0.001
2016–2019	0.50 (0.41–0.61)	<0.001

Odds ratio (95% CIs) for each time period versus period A (reference) obtained from logistic regression model adjusted for the following variables: mitral valve repair: age, BMI, sex, EF range, NYHA score, CCS score, MV stenosis, MV pathology, coronary disease, hypertension, previous stroke/TIA, previous MI, redo sternotomy, PVD, urgency, concomitant ablation, concomitant CABG, concomitant TV surgery, cross-clamp times, cardiopulmonary bypass times. Mitral valve replacement: age, BMI, sex, smoking status, EF range, CCS score, MV stenosis, MV pathology, diabetes, coronary disease, hypertension, COPD, previous stroke/TIA, redo sternotomy, AF/flutter, cardiopulmonary bypass time, cross-clamp time, urgency, preoperative dialysis, concomitant ablation, concomitant CABG, concomitant TV surgery.

AF: atrial fibrillation; BMI: body mass index; CABG: coronary artery bypass graft; CCS: Canadian Cardiovascular Society; CI: confidence interval; COPD: chronic obstructive pulmonary disease; EF: ejection fraction; MI: myocardial infarction; MV: mitral valve; NYHA: New York Heart Association; PVD: peripheral vascular disease; TIA: transient ischaemic attack; TV: tricuspid valve.

## DISCUSSION

Our results demonstrate that MV surgery in the UK has shifted towards older, more comorbid patients. These trends are in keeping with findings in the US based on nationally collected data, which demonstrate a similar shift towards higher levels of preoperative risk [1]. An interesting divergence between our findings and the US data is their increasing proportion of patients presenting with severe left ventricular dysfunction [1]. This suggests that UK guidelines are performing well in prioritizing early identification and referral of suitable candidates for MV surgery. This is further supported by the trends in NYHA scores in both groups which show patients are less likely to present with severe symptom profiles. An alternative interpretation of these results is that sicker patients may be more likely to receive surgery in the US compared to the UK.

The proportion of MVr patients presenting as redo MV surgery rose significantly over time; however, they remained stable in patients undergoing MVR. The overall rise in redo cases is not surprising given the ageing population and the increasing number of patients with good long-term survival after index procedure. It is also likely a reflection of growing confidence over time resulting in a greater propensity to take on more difficult, complex redo cases and repair them where possible.

In terms of intraoperative variables, both CPB time and cross-clamp time increased steadily over time before dropping in the latest time period. The trend may represent the MV surgical learning curve in the UK, as well as the demographic shifts over time. Patients are presenting increasingly older and more comorbid, therefore requiring more challenging operations. The rise of minimally invasive surgery, which is not captured in this dataset, may also contribute to this observation. We have shown that MVR consistently requires longer CPB and AoX times than MVr.

Our results confirm degenerative disease as the most common indication for both MVr and MVR^2^. It is encouraging that repair rates for degenerative disease have doubled from 38% in period A to 76% in period E. When comparing against the US data, our repair rates for degenerative disease remain lower than the rate of 80.3% quoted in a large US analysis of 55 311 patients between July 2011 and December 2016 [12]. The trend towards repair reflects the growing acceptance of MVr as the gold standard for degenerative disease [5]. An opposite trend was observed in ischaemic disease, with the overall repair rate dropping significantly from 78% in period C to 54% in period E, likely in response to evidence published in 2014 demonstrating that MVR provided a more durable correction of ischaemic mitral regurgitation, with no significant between-group differences in clinical outcomes [4].

Our analyses show that acute endocarditis as an indication for MVr is rising again in keeping with current literature [13, 14]. Whilst there has been an increase in repair rates for acute endocarditis, they remain lower than for degenerative and ischaemic disease, reflecting the associated technical difficulty (see Fig. 1). Despite this, there have been encouraging studies supporting excellent outcomes for repair in endocarditis if performed early in experienced centres, with repair rates as high as 81% quoted in one European study [15]. Given the increasing burden of MV endocarditis, further efforts to optimize early referral for surgery and encourage repair strategies may result in improved long-term outcomes.

Rates of AF ablation have risen significantly over time in both groups, more significantly in patients undergoing MVr. As a rhythm control strategy, the superiority of surgery with concomitant Maze procedure over surgery alone with medical rhythm control has been demonstrated [16]. The higher level of comorbidities as well as more challenging pathologies associated with patients undergoing MVR, as well as the generally longer operative times, may explain the discrepancy in AF ablation rates. The learning curve required may also pose a barrier to generally higher rates of AF ablation in the overall MV cohort.

Trends in operative urgency differed between MVr and MVR groups. Over time, MVr patients were more likely to present as elective cases; patients undergoing MVR were more likely to be in an urgent or emergency context. Unadjusted outcome trends are encouraging, with mortality dropping significantly in both groups. The drop was more appreciable in MVr patients, with mortality more-than-halving from 5% to 2% compared to a smaller decrease from 9% to 7% in MVR. This observed reduction in mortality was also found after adjusting for confounding variables. Improvements were also observed in secondary outcome trends, with significant reductions in rates of stroke and re-exploration for bleeding in both cohorts. Rates of postoperative dialysis improved in MVr patients over time but remained stable in patients receiving MVR. Over time the lengths of admission improved in MVr but remained the same for MVR.

**Figure 2:**
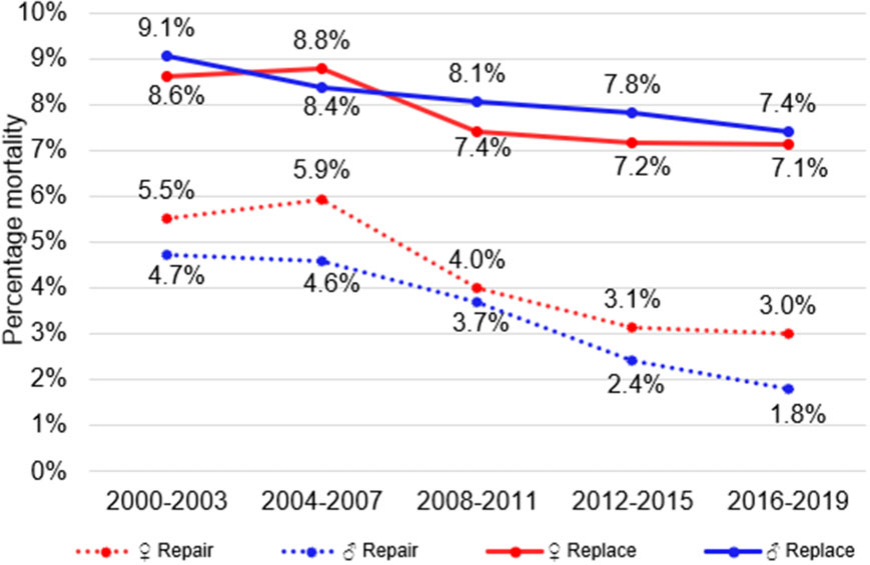
Mortality rates by sex and procedure over time.

**Figure 3:**
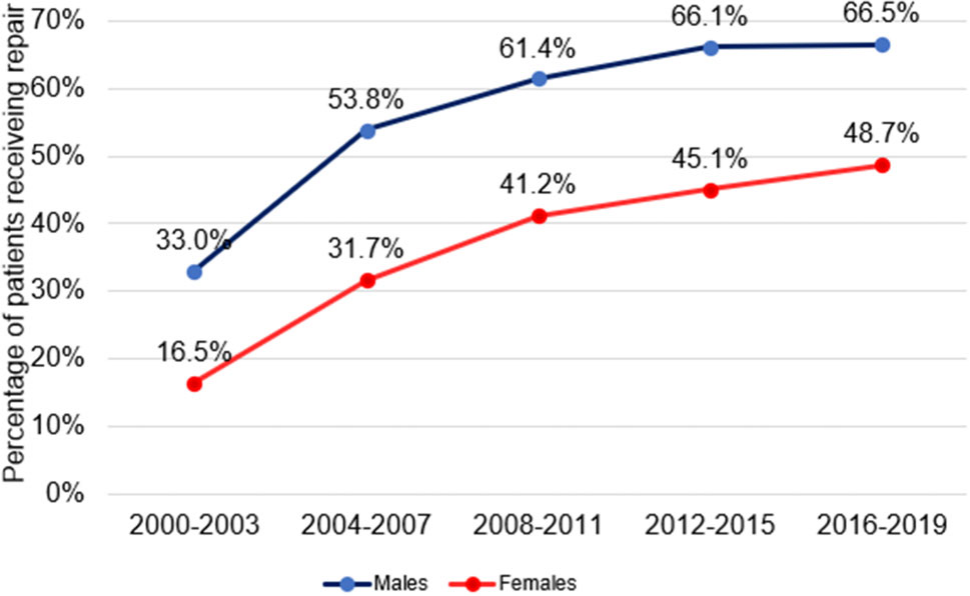
Mitral repair rates over time in males and females.

When looking at overall mortality and repair trends in males and females (see Figs 2 and 3), males experience superior outcomes compared to females. Repair rates remained persistently higher in males than females (Fig. 3), with significantly lower mortality rates in males receiving MVr compared to females. Differing pathologies between men and women have been previously reported, with women more likely to suffer from anterior leaflet disease which is less amenable to repair [17]. Whilst this partially explains our findings, it is important to consider the possibility of practitioner bias contributing to these outcomes, such as later referral of females for MVS compared and/or less effective repair techniques in females. Our research team is undertaking further detailed research looking specifically at sex-based discrepancies to explain better why they exist and how to alleviate them.

The strengths of our analyses include the large sample from a national audit, making our findings reasonably generalizable to the UK population. In most variables, there was a low degree of missingness. Our conclusions need to be interpreted in light of limitations associated with a nonrandomized, retrospective study design. Whilst the NACSA data are regularly maintained and validated, it is possible that some data may have been reported incorrectly. Further granularity into the specific mitral lesions, repair techniques used and whether a repair was attempted before repair would have allowed for a more rigorous analysis. Unfortunately, we did not have access to data on patient ethnicity, the mode of surgical incision or rates of postoperative permanent pacemaker implantation. These are important trends to capture going forward, particularly as the adoption of minimally invasive techniques continues to rise. Finally, we did not have data on long-term outcomes (e.g. mortality, stroke, hospital readmission) because the NACSA dataset is not linked with hospital episode or mortality data.

## CONCLUSIONS

1. Overall mortality has dropped significantly in both in MVr and in MVR.
2. The latest national repair rate for degenerative disease is 76%.
3. Females have worse outcomes than males, with higher mortality and lower repair rates.
4. MVr has overtaken MVR as the most common procedure and is also increasing in patients with endocarditis.

## Supplementary Material

ivad086_Supplementary_Data

## Data Availability

The data underlying this article were provided by NICOR by permission. Data will be shared on request to the corresponding author with permission of NICOR. **Fadi Al-Zubaidi:** Conceptualization; Investigation; Methodology; Project administration; Visualization; Writing—original draft; Writing—review & editing. **Maria Pufulete:** Conceptualization; Formal analysis; Methodology; Supervision; Writing—review & editing. **Shubhra Sinha:** Data curation; Investigation; Writing—review & editing. **Simon Kendall:** Supervision; Writing—review & editing. **Narain Moorjani:** Supervision; Writing—review & editing. **Gianni D. Angelini:** Data curation; Funding acquisition; Investigation; Supervision; Writing—review & editing. **Massimo Caputo:** Data curation; Supervision; Writing—review & editing. **Hunaid A. Vohra:** Conceptualization; Data curation; Formal analysis; Investigation; Methodology; Supervision; Writing—review & editing. Interactive CardioVascular and Thoracic Surgery thanks the anonymous reviewer(s) for their contribution to the peer review process of this article.

## ABBREVIATIONS

AF: Atrial fibrillation
AoX: Aortic cross-clamp
CIs: Confidence intervals
CPB: Cardiopulmonary bypass
MVr: Mitral valve repair
MVR: Mitral valve replacement
MVS: Mitral valve surgery
NACSA: National Cardiac Surgery Activity and Outcomes Report
NICOR: National Institute for Cardiovascular Outcomes Research
NIHR: National Institute for Health Research
OR: Odds ratios
