# Supplementary material for: Mitral repair versus replacement: 20-year outcome trends in the UK (2000–2019)
Source: Interdiscip Cardiovasc Thorac Surg. 2023 May 19;36(6):ivad086. doi: 10.1093/icvts/ivad086 (PMC10250075; doi:10.1093/icvts/ivad086)
Supplement: ivad086_Supplementary_Data [file ivad086_supplementary_data.pdf]

Web Appendices

Intel Inside: The Linguistic Properties of Effective Slogans

Brady T. Hodges

Zachary Estes

Caleb Warren

A. Slogan Dataset Collection, Study 1 (p. 3)
B. Additional Analyses, Study 1
    I. Additional Variables: Full Model (p. 4)
    II. Model Reduction (p. 6)
    III. Variable Selection (p. 7)
    IV. Factor Reduction (p. 9)
    V. Recognition Memory: Hits and False Alarms (p. 12)
    VI. Non-average Models (p. 13)
    VII. Robustness Test: Brand Attitude (p. 15)
    VIII. Online Replication and Combined Analysis (p. 16)
    IX. Interaction Model with Brand Familiarity (p. 17)
C. Slogan Stimuli in Studies 2-4 (p. 19)
D. Linguistic Fluency Validation Measure (p. 23)
E. Memory and Liking of Individual Slogans (p. 25)
F. Study 2 Replication—Brand Name Repetition (p. 29)
G. Study 4b Replication—Slogan Recall (p. 31)

Web Appendix A: Slogan Dataset Collection, Study 1

We collected a large dataset of actual brand slogans for analysis in Study 1 from the public repositories of various online slogan consultancy firms and websites. The initial collection resulted in 1,243 brand slogans, of which 830 brand slogans were unique. We then collected liking, memory, and familiarity ratings, as described in Study 1.

Lewis Silkin – AdSlogans Search Database
https://www.lewissilkin.com/en/cmi/services/intellectual-property/adslogans

Eric Swartz – TaglineGuru (The Byline Group Inc.) Database
https://www.taglineguru.com/sloganlist.html

Adglitz – Online Slogan Repository
-http://www.adglitz.com/blog/2010/08/top-n-best-100-ad-slogans-taglines-punchlines-advertising-campaigns
-http://www.adglitz.com/blog/2011/01/top-n-best-100-ad-slogans-taglines-punchlines-advertising-campaigns-part-2-of-2

Online Slogan Repositories
-http://www.workplace-communication.com/top-100-slogans.html
-http://www.ad-mad.com/slogans

Web Appendix B: Additional Analyses, Study 1

I. Additional Variables: Full Model

In the main text we present results of a "linguistic model" (Table 1B) that included five key linguistic properties that we found to predict slogan liking and/or memory. As is standard in psycholinguistic research (e.g., Balota et al. 2004; Kuperman et al. 2014), however, we also analyzed a host of additional factors known to influence word processing. Here we describe the full set of possible predictor variables that we examined, and in subsequent sections of Web Appendix B we explain how we selected those five key variables included in our "linguistic model," we describe alternative methods for selecting variables, and we report comparisons to other possible models.

In total, we examined fifteen lexical, semantic, and affective variables for each slogan. We measured *slogan length* in two ways, as the number of (i) words and (ii) characters in the slogan. We used four measures to assess *word length*: the average number of (i) letters, (ii) phonemes, (iii) syllables, and (iv) morphemes of the words in the slogan (Balota et al. 2007). We used two measures to assess how *frequent* each word is in language: (i) the number of times the word appears in the SUBTLEX-US corpus (i.e., word frequency; Brysbaert and New 2009), and (ii) the number of different semantic contexts in which the word occurs in the SUBTLEX-US corpus (i.e., contextual diversity; Adelman, Brown, and Quesada 2006). As is standard, we log-transformed the frequency measures to correct for skew. We determined the *perceptual distinctiveness* of the words using both orthographic Levenshtein distance, which measures visual distinctiveness, and phonological Levenshtein distance, which measures auditory distinctiveness (Balota et al. 2007). We obtained values for two semantic properties of the words: *age-of-acquisition* and *concreteness*, from the databases collected by Kuperman, Stadthagen-Gonzalez, and Brysbaert (2012) and Brysbaert, Warriner, and Kuperman (2014), respectively. We retrieved two affective properties of the words: *arousal* and *valence*, from the database collected by Warriner, Kuperman, and Brysbaert (2013). Finally, we created a *brand name* variable by dummy coding whether the brand name was present in the slogan (1) or not (0).

We regressed slogan liking and slogan memory on all fifteen lexical, semantic, and affective variables, plus slogan familiarity (as a control factor). Note that because affective scores (i.e., arousal and valence) were not available for 28 of the slogans, analyses that include these factors have $N = 792$ slogans. As shown in Table A1, the full model explained a significant amount of variance in both slogan liking, $R^2 = 0.52$, $F(16, 775) = 51.65$, $p < .001$, and slogan memory, $R^2 = 0.25$, $F(16, 775) = 16.18$, $p < .001$.

Perhaps unsurprisingly, slogan familiarity significantly predicted both slogan liking ($b = 0.47$, SE = 0.02, $t = 25.52$, $p < .001$) and slogan memory ($b = 1.45$, SE = 0.31, $t = 4.62$, $p < .001$). Of the two measures of slogan length, number of words predicted both slogan liking ($b = 0.11$, SE = 0.04, $t = 3.02$, $p = .003$) and slogan memory ($b = 1.32$, SE = 0.62, $t = 2.14$, $p = .033$), whereas number of characters predicted liking ($b = -0.02$, SE = 0.01, $t = 3.28$, $p < .001$) but not memory ($b = 0.00$, SE = 0.11, $t = 0.03$, $p = .973$). None of the four measures of word length predicted either liking or memory of the slogans. Of the two measures of word frequency, contextual diversity significantly predicted slogan liking ($b = 0.42$, SE = 0.20, $t = 2.05$, $p = .041$) and marginally predicted slogan memory ($b = -6.57$, SE = 3.48, $t = 1.89$, $p = .060$). Of the two measures of perceptual distinctiveness, orthographic (visual) distinctiveness marginally predicted slogan liking ($b = 0.36$, SE = 0.19, $t = 1.89$, $p = .059$), whereas phonological (auditory) distinctiveness did not predict liking or memory. Concreteness significantly predicted both slogan liking ($b = -0.13$, SE = 0.06, $t = 2.14$, $p = .033$) and slogan memory ($b = 3.94$, SE = 1.00, $t = 3.93$, $p < .001$). Age of acquisition, arousal, and valence

had no effect on either liking or memory. Finally, brand name inclusion significantly predicted both slogan liking (*b* = -0.33, SE = 0.06, *t* = 5.82, *p* < .001) and slogan memory (*b* = 6.80, SE = 0.97, *t* = 7.04, *p* < .001).

Table A1. Results of the full model.

	Dependent Variable	
Predictor	(1) Slogan Liking	(2) Slogan Memory
Control: Slogan Familiarity[a]	0.47*** (0.02)	1.45*** (0.31)
Slogan Length		
Number of Words[a]	0.11** (0.04)	1.32* (0.62)
Number of Characters	-0.02*** (0.01)	0.00 (0.11)
Word Length		
Number of Letters	0.08 (0.08)	-2.23 (1.37)
Number of Phonemes	-0.06 (0.09)	-0.59 (1.50)
Number of Syllables	-0.20 (0.14)	1.84 (2.44)
Number of Morphemes	0.10 (0.14)	-1.88 (2.35)
Word Frequency		
Word Frequency	-0.15 (0.13)	-2.63 (2.19)
Contextual Diversity[a]	0.42* (0.20)	-6.57† (3.48)
Perceptual Distinctiveness		
Orthographic[a]	0.36† (0.19)	1.76 (3.22)
Phonological	0.04 (0.15)	1.87 (2.63)
Age of Acquisition	0.04 (0.04)	-0.47 (0.59)
Concreteness[a]	-0.13* (0.06)	3.94*** (1.00)
Arousal	0.00 (0.04)	-0.31 (0.71)
Valence	0.05 (0.03)	-0.76 (0.53)
Brand Name[a]	-0.33*** (0.06)	6.80*** (0.97)
Intercept	1.78** (0.68)	111.90*** (11.56)
R^2	0.52***	0.25***

Note. Unstandardized coefficients; standard errors appear in parentheses. Slogan Liking was rated on a scale from 1 (dislike) to 7 (like); Slogan Memory is % correct (Hits – False Alarms). † *p* < .06, * *p* < .05, ** *p* < .01, *** *p* < .001.
[a]Variable selected for inclusion in the “linguistic model.”

II. Model Reduction

As described above, the full model included fifteen linguistic predictors (plus slogan familiarity). Models with many variables tend to face two fundamental limitations, one conceptual and one statistical. Conceptually, models with many predictor variables tend to be theoretically complex and difficult to interpret. Statistically, models with many predictor variables tend to exhibit multicollinearity. Indeed, our full model included multiple measures of some variables. For instance, we measured word length in four ways: number of letters, number of syllables, number of phonemes, and number of morphemes. We also had two measures of slogan length (number of words, number of characters), two measures of word frequency (prevalence, diversity), and two measures of word distinctiveness (visual, auditory). Although this approach maximizes the variance explained (R^2), it can also distort the effect sizes of the individual predictors due to their inherent multicollinearity. For example, because the four different measures of word length are naturally and highly intercorrelated, that multicollinearity can artificially suppress the effect size of all four individual measures, potentially obscuring effects that otherwise would be significant.

To address these limitations of conceptual complexity and multicollinearity, a common practice when analysing the effects of a large number of predictor variables is to simplify the model by reducing the number of predictors. There are several methods for model reduction. One general approach (i.e., *variable selection*) is to simply select the best predictors and exclude all predictors that add little or no predictive value to the model. We performed two such analyses. The simplest approach is to enter all the individual predictors simultaneously into a standard OLS regression (i.e., the full model reported above), and retain only those variables that significantly predict slogan liking and/or memory (i.e., *significant predictors*). This corresponds to the "linguistic model" that we report in the main text. An alternative and more sophisticated method for simultaneously reducing collinearity and identifying the most predictive variables is *penalized regression*, such as Lasso and Ridge, which essentially penalize regression models that have too many predictor variables.

A second general approach (i.e., *factor reduction*) is to identify latent factors that combine several of the previously separate predictors and create weighted factor scores for each latent factor (e.g., via principal components analysis, or *PCA*). We again performed two such analyses. First, in an *unconstrained* factor-analytic model, all fifteen linguistic variables were simultaneously entered into the same PCA without any constraints. An alternative and more constrained approach is to specifically combine only the various measures of each linguistic variable. That is, in the *constrained* factor-analytic model, we conducted separate PCAs for each variable that had multiple measures (i.e., slogan length, word length, word frequency, and perceptual distinctiveness).

Thus, below we report four methods of model reduction: (i) selection of significant predictors, (ii) penalized regression, (iii) unconstrained factor analysis, and (iv) constrained factor analysis. We also statistically compared these four different methods, with the goal of identifying the model that best predicted slogan liking and memory with the fewest variables. In short, the best method was the "linguistic model" that retained only five linguistic predictors. This linguistic model converged with the results of the Lasso and Ridge penalized regressions, and it also significantly outperformed both the unconstrained and constrained factor-analytic models in predicting both slogan liking and slogan memory.

III. Variable Selection

A. Significant Predictors: Linguistic Model

Results of the full model (Table A1) reveal that whereas some of the individual linguistic variables are important for predicting slogan liking and memory, others appear less predictive. For theoretical parsimony, and to reduce multicollinearity among the variables, we selected for presentation in the main text ("linguistic model") only those linguistic variables (in addition to slogan familiarity) that significantly or marginally predicted either slogan liking or slogan memory. Specifically, we selected (1) slogan length (number of words), (2) word frequency (contextual diversity), (3) perceptual distinctiveness (orthographic), (4) concreteness, and (5) inclusion of the brand name. We included number of words instead of number of characters as our measure of slogan length because the former significantly predicted both liking and memory, whereas the latter only predicted liking and not memory.

To compare the "full model" of all fifteen linguistic variables (Table A1) to the "linguistic model" of the five selected linguistic variables (Table 1B of main text), we conducted a stepwise regression with slogan familiarity and the five selected variables entered in a first block and the remaining ten non-selected linguistic variables (Table A1) entered in a second block. Results revealed that adding the ten variables increased the variance explained by only 1.1%, from 50.5% in the linguistic model to 51.6% in the full model. This difference was only marginally significant, $F(10, 775) = 1.78$, $p = .061$, indicating that the linguistic model represented a negligible loss of predictive power despite the large decrease in model complexity. A stepwise regression on slogan memory yielded similar results: Relative to the linguistic model ($R^2 = 23.7\%$), the full model increased the variance explained by only 1.3% ($R^2 = 25.0\%$), despite including ten additional factors. This difference was not significant, $F(10, 775) = 1.33$, $p = .21$, indicating no loss of predictive power for the linguistic model.

B. Penalized Regression: Lasso and Ridge

Lasso regression uses L1 regularization to set the weighting of less predictive variables to zero, thereby effectively selecting the subset of predictors that are most important for explaining the variance in the dependent variable. Rather than setting those less predictive variables to zero, Ridge regression instead retains all predictors but uses L2 regularization to shrink less predictive ones toward zero. Thus, whereas Lasso excludes the less important predictors from the model, Ridge merely minimizes their weighting. And conversely, factors that are retained in Lasso and that have coefficients in Ridge that diverge from zero are those identified as important for the predictive accuracy of the model.

We therefore conducted Lasso and Ridge regressions on slogan liking and slogan memory, as a robustness test of the variable selection in our "linguistic model" in the main text (cf. Packard and Berger 2021). If our five key linguistic variables are retained in the Lasso models, and if their coefficients do not approach zero in the Ridge models, this would suggest that those variables are important for predicting slogan liking and/or slogan memory. We used the glmnet package in R, with an 80% training set and 20% hold-out sample, and we used cross-validation to identify the optimal value for lambda. See Table A2 for results.

Overall, there was little difference between Lasso and Ridge in terms of amount of deviance explained (interpretable as R^2), and both were comparable to the variance explained by the full model (see Table A1). More importantly, both Lasso and Ridge regressions indicate that all five of the selected linguistic variables (i.e., "Linguistic Model" in Table A2) are important for predicting slogan liking and/or slogan memory. In Lasso, each of the five

linguistic variables was retained in the model predicting either liking or memory. Critically, nearly all of the other linguistic variables were dropped from the Lasso models, indicating that any increase in predictive accuracy that they might provide would not justify the increase in model complexity that they would require. Similarly, in Ridge, the coefficients of our five key linguistic properties tended to diverge substantially from zero. Thus, our linguistic model was robust to penalized regression.

Table A2. Results of penalized regression models.

	Dependent Variable			
	Slogan Liking		Slogan Memory	
Predictor	(1) Lasso	(2) Ridge	(3) Lasso	(4) Ridge
Control: Slogan Familiarity	0.46	0.44	1.19	1.27
Linguistic Model				
Slogan Length (words)		0.03	1.25	0.84
Word Frequency (diversity)	0.13	0.20	-8.07	-6.22
Distinctiveness (visual)	0.14	0.24		0.83
Concreteness	-0.12	-0.11	4.08	3.73
Brand Name	-0.34	-0.33	6.49	6.28
Additional Variables				
Slogan Length (characters)	-0.002	-0.01		0.08
Word Length (letters)		0.02	-0.68	-1.43
Word Length (syllables)		-0.12		1.02
Word Length (phonemes)		-0.02		-0.61
Word Length (morphemes)		0.06	-0.93	-1.88
Word Frequency (frequency)		-0.002		-1.88
Distinctiveness (auditory)		0.04		1.13
Age of Acquisition		0.03		-0.36
Arousal		0.01		-0.22
Valence	0.03	0.05	-0.54	-0.79
Intercept	2.83	2.26	99.55	108.65
Deviance (R^2)	0.506	0.510	0.243	0.247

Note. Unstandardized coefficients. Slogan Liking was rated on a scale from 1 (dislike) to 7 (like); Slogan Memory is % correct (Hits – False Alarms).

Interestingly, although valence was not a significant predictor in the standard OLS regressions of the full model (see Table A1), these penalized regressions indicate that this variable could be important for predicting both liking and memory of slogans. Specifically, as with brand names (Guest, Estes, Gibbert, and Mazursky 2016), slogans with more positive words appear to be liked more but remembered less. The valence of the words within a slogan thus may be a fruitful topic for further research.

IV. Factor Reduction

A. Unconstrained Factor-Analytic Model

We submitted the fifteen lexical, semantic, and affective properties to a PCA with Varimax rotation. The analysis identified four latent factors with eigenvalue greater than 1. Rotated factor loadings are shown in Table A3. The first factor, which included mostly lexical properties (i.e., word length, word frequency, distinctiveness, and age of acquisition), accounted for 45.41% of the variance among the predictors' scores. The second factor corresponded solely to slogan length and explained an additional 13.51% of variance. The third factor consisted of concreteness and the two affective properties (i.e., arousal and valence) and explained 9.56% of residual variance. Finally, the fourth factor loaded on brand name inclusion, explaining 7.16% of residual variance. Collectively the four latent factors explained 75.64% of the variance in the fifteen properties.

Table A3. Factor loadings.

Property	Factor 1	Factor 2	Factor 3	Factor 4
Slogan Length (Words)	-0.25	**0.94**	-0.02	0.06
Slogan Length (Characters)	0.03	**0.97**	-0.03	0.09
Word Length (Letters)	**0.94**	-0.09	0.00	-0.05
Word Length (Syllables)	**0.92**	-0.04	0.10	-0.07
Word Length (Phonemes)	**0.95**	-0.09	0.00	-0.03
Word Length (Morphemes)	**0.82**	0.00	0.01	-0.02
Word Frequency (Prevalence)	**-0.78**	0.22	0.33	-0.31
Word Frequency (Diversity)	**-0.75**	0.17	0.32	-0.34
Distinctiveness (Visual)	**0.91**	-0.01	0.11	-0.09
Distinctiveness (Aural)	**0.91**	-0.03	0.13	-0.10
Age of Acquisition	**0.73**	-0.10	0.00	0.06
Concreteness	-0.02	-0.13	**-0.73**	0.28
Arousal	0.21	-0.13	**0.45**	0.41
Valence	-0.06	-0.11	**0.66**	0.19
Brand Name	-0.13	0.20	0.01	**0.74**

We then regressed slogan liking on these four weighted factor scores plus slogan familiarity. As shown in Table A4, the overall model was significant, $F(5, 786) = 156.51$, $p < .001$, and explained 50% of the variance in slogan liking. In addition to slogan familiarity ($b = 0.47$, SE = 0.02, $t = 25.45$, $p < .001$), factors 3 (concreteness, arousal, and valence; $b = 0.10$, SE = 0.02, $t = 4.26$, $p < .001$) and 4 (brand name; $b = -0.16$, SE = 0.02, $t = 6.52$, $p < .001$) significantly predicted liking: Slogans that use words that are less concrete, more arousing, and more positive, and that exclude the brand name, tend to be liked more. (Note: the loading of concreteness on factor 3 is negative, so the positive coefficient of factor 3 indicates a negative relation between concreteness and liking.)

We also regressed slogan memory on these four factors plus slogan familiarity. As shown in Table A4, the overall model was significant, $F(5, 786) = 46.64$, $p < .001$, and explained 23% of the variance in memory accuracy. In addition to slogan familiarity ($b = 1.39$, SE = 0.31, $t = 4.44$, $p < .001$), factors 2 (slogan length; $b = 3.57$, SE = 0.42, $t = 8.50$, $p < .001$), 3 (concreteness, arousal, and valence; $b = -3.14$, SE = 0.41, $t = 7.60$, $p < .001$), and 4

(brand name; $b = 4.19$, SE = 0.41, $t = 10.12$, $p < .001$) significantly predicted slogan memory. Slogans that are longer, that include the brand name, and that use concrete words (that are low in arousal and valence) tend to be more memorable. These factor-analytic results are generally consistent with those of the "linguistic model" reported in the main text.

Table A4. Results of unconstrained factor-analytic model.

	Dependent Variable	
Predictor	(1) Slogan Liking	(2) Slogan Memory
Control: Slogan Familiarity	0.47*** (0.02)	1.39*** (0.31)
Factor 1	0.03 (0.02)	0.05 (0.41)
Factor 2	-0.04 (0.03)	3.57*** (0.42)
Factor 3	0.10*** (0.02)	-3.14*** (0.41)
Factor 4	-0.16*** (0.02)	4.19*** (0.41)
Intercept	3.25*** (0.05)	83.54*** (0.87)
R^2	0.50***	0.23***

Note. Unstandardized coefficients; standard errors appear in parentheses. Slogan Liking was rated on a scale from 1 (dislike) to 7 (like); Slogan Memory is % correct (Hits – False Alarms). *** $p < .001$.

Finally, we compared this unconstrained factor-analytic model (Table A4) to the linguistic model with the five individual linguistic properties reported in the main text (Table 1B). We conducted a stepwise regression, with slogan familiarity and the four latent factors (Table A4) entered in a first block and the five individual linguistic properties (Table 1B, linguistic model) entered in a second block. The linguistic model significantly outperformed the unconstrained factor-analytic model in predicting both slogan liking, $\Delta R^2 = .014$, $F(5, 781) = 4.33$, $p < .001$, and slogan memory, $\Delta R^2 = .015$, $F(5, 781) = 3.18$, $p = .008$.

In sum, results of these analyses (i.e., with four unconstrained latent factors) generally converged with those reported in the main text (i.e., with five linguistic properties). Further, the linguistic model in the main text outperformed this factor-analytic model in predicting both slogan liking and slogan memory.

B. Constrained Factor-Analytic Model

For each variable that was measured in multiple ways, we submitted all measures of that variable to PCA (with Varimax rotation), and crucially, we then used only the weighted factor score to represent that variable in our statistical model. For example, we used PCA to create a weighted index of the four measures of word length, thereby reducing that variable to a single measure. We similarly created weighted indexes of slogan length, word frequency, and word distinctiveness. Each of these four PCAs yielded a single latent factor (eigenvalue > 1), confirming the reliability of the various measures within each factor.

For each variable that we originally measured via only a single measure (i.e., brand name, age of acquisition, concreteness, arousal, and valence), we retained those single measures in the analysis. Thus, in this analysis, the original fifteen lexical, semantic, and affective variables were reduced to nine variables, each with a single measure. We then regressed slogan liking and slogan memory on these nine variables, plus slogan familiarity. Results are shown in Table A5.

Table A5. Results of constrained factor-analytic model.

Predictor	Dependent Variable (1) Slogan Liking	(2) Slogan Memory
Control: Slogan Familiarity	0.47*** (0.02)	1.43*** (0.31)
Slogan Length (factor)	-0.03 (0.03)	3.77*** (0.43)
Word Length (factor)	-0.06 (0.06)	-3.68*** (1.06)
Word Frequency (factor)	0.08 (0.04)	-4.38*** (0.74)
Distinctiveness (factor)	0.11 (0.06)	1.64 (0.95)
Age of Acquisition	0.03 (0.03)	-0.55 (0.58)
Concreteness	-0.12* (0.06)	3.41*** (0.98)
Arousal	0.01 (0.04)	-0.25 (0.70)
Valence	0.05 (0.03)	-0.86 (0.53)
Brand Name	-0.36*** (0.06)	6.88*** (0.95)
Intercept	3.22*** (0.39)	81.30*** (6.70)
R^2	0.51***	0.24***

Note. Unstandardized coefficients; standard errors appear in parentheses. Slogan Liking was rated on a scale from 1 (dislike) to 7 (like); Slogan Memory is % correct (Hits – False Alarms). * $p < .05$; *** $p < .001$.

Finally, we compared this constrained factor model (Table A5) to the linguistic model (Table 1B). We conducted a stepwise regression, with slogan familiarity and the nine constrained factors (Table A5) entered in a first block and the five individual linguistic variables (Table 1B) entered in a second block.[1] The linguistic model significantly outperformed the constrained factor model in predicting slogan liking, $\Delta R^2 = .007$, $F(3, 778) = 3.79$, $p = .010$. The difference in predicting slogan memory was directional but nonsignificant, $\Delta R^2 = .006$, $F(3, 778) = 2.02$, $p = .109$.

Thus, results of this constrained factor analysis (i.e., with nine single-measure factors) generally converged with those reported in the main text (i.e., with five linguistic properties). Further, that linguistic model in the main text outperformed this constrained factor-analytic model in predicting slogan liking.

In sum, of the four approaches to model reduction, simply selecting the significant predictors from the full model is the most theoretically parsimonious approach, while also providing the most accurate prediction of slogan liking and slogan memory. This approach, which we call the “linguistic model” in the main text, was robust to both Lasso and Ridge penalized regression, and it significantly outperformed both the unconstrained and the constrained factor-analytic models. Therefore, in all subsequent analyses reported below, we use only this linguistic model.

[1] Because the brand name and concreteness variables were measured via a single item each, entering them in both the first block and the second block was redundant, so actually the second block added only slogan length (number of words), word frequency (diversity), and distinctiveness (visual).

V. Recognition Memory: Hits and False Alarms

In the main text we report analyses of *memory accuracy*, which is calculated by subtracting each slogan's false alarm rate from its hit rate (Cortese, Khanna, and Hacker 2010). The *hit rate* is the percentage of participants who correctly reported that they had seen the slogan after having actually seen it. The *false alarm rate* is the percentage of participants who incorrectly reported that they had seen the slogan despite having not actually seen it. Here we report results of the hit rate and the false alarm rate separately (Table A6).

Table A6. Results of hit rates and false alarm rates.

	Dependent Variable	
Predictor	(1) Hits	(2) False Alarms
Control: Slogan Familiarity	1.52*** (0.25)	0.18 (0.16)
Linguistic Variable		
Slogan Length	1.19*** (0.12)	-0.16* (0.08)
Word Frequency	-6.24*** (1.06)	1.50* (0.68)
Distinctiveness	-1.25 (1.06)	0.19 (0.68)
Concreteness	3.44*** (0.69)	-0.71 (0.44)
Brand Name	7.44*** (0.74)	0.13 (0.48)
Intercept	91.73*** (5.75)	-0.41 (3.69)
R^2	0.28***	0.02*

Note. Unstandardized coefficients; standard errors appear in parentheses. * $p < .05$; *** $p < .001$.

In sum, the linguistic factors that significantly predicted overall memory accuracy (Table 1B of main text) also predicted hit rates better than false alarm rates (Table A6). Thus, the effects observed on overall memory accuracy are largely attributable to hits rather than false alarms.

VI. Non-Average Models

The analyses that we present in the main text are based on measures that are averaged across individual words in the slogan. For instance, for the slogan "Just do it", we retrieved the linguistic properties (e.g., word frequency) of "just", "do", and "it", and then we averaged across the three words of the slogan to create an *average* value for each property (e.g., an *average* word frequency). But of course, averaging is not the only way to examine these properties, and although averaging does examine the linguistic properties of *individual* words, it does not examine the properties of *single* words.

We therefore replicated our analyses, but using alternative, non-average measures. These alternative models provided a test of whether the "average" model (i.e., the model in which the linguistic properties are averaged across words within the slogan) is actually the best model for predicting slogan liking and/or memory. First we tested a "Max" model that took as its value for each predictor the maximum value among all words in the slogan. For instance, given that "it" is the most frequent word in "Just do it", we used the frequency of "it" as the measure of word frequency for that slogan. For visual distinctiveness, however, we used the value of "just", because it was the most distinctive word in the slogan. This model tested whether the *average frequency* (and length, and concreteness, etc.) of words in the slogan best predicts liking and memory, or alternatively whether the *single most frequent* word is a better predictor. We similarly created a "Min" model by taking, for each linguistic variable, the minimum value among all words in the slogan.

Or perhaps rather than the average, max, or min, the *range* of values best predicts slogan liking and memory. That is, perhaps having some words stand out from other words within the slogan is especially effective. To test this, we also created a "Range" model that took as its values the range (i.e., max – min) of values for each linguistic variable. In this model, higher numbers indicate greater variation on the given linguistic variable within the slogan. Finally, we note that some of the linguistic properties are predicted to increase liking (and decrease memory), whereas other properties are predicted to decrease liking (and increase memory). We therefore created a "MiniMax" model that took as its values the maximum value within the slogan of word frequency and visual distinctiveness, but took the minimum value of concreteness.

We conducted standard OLS regressions for each type of model (i.e., average, max, min, range, minimax). Note that because slogan familiarity, slogan length, and brand name are all slogan-level variables, only word frequency, visual distinctiveness, and concreteness varied across the models. Results are summarized in Table A7.

Table A7. Comparison of average and non-average models.

	**Liking**		**Memory**	
	F	R^2	***F***	R^2
**Average**	**137.54**	**0.504**	**40.36**	**0.229**
Max	133.76	0.493	36.18	0.211
Min	135.87	0.501	34.44	0.203
Range	134.16	0.498	35.93	0.210
MiniMax	135.57	0.500	25.25	0.157

Note. DF = 6, 813. All models were $p < .001$.

As shown above, although the differences among models were small, the "average" model (i.e., the model in which the linguistic properties are averaged across words within the

slogan) was the best performer for both slogan liking and slogan memory. In other words, averaging the linguistic properties across multiple words within the slogan better predicts the slogan's liking and memory than using measures based on a single word within the slogan (e.g., the most frequent word, or the least concrete word).

VII. Robustness Test: Brand Attitude

To test the robustness of the results of Study 1, we replicated the linguistic model, but with one alternative predictor variable and one alternative dependent variable. First, in Study 1 we measured not only *slogan familiarity* ("Prior to this survey, how familiar were you with this slogan?"), which is reported in the main text, but also *brand familiarity* ("Prior to this survey, how familiar were you with this brand?"). In this robustness test, we replaced slogan familiarity with brand familiarity. Second, in Study 1 we also measured not only *slogan liking* ("Overall, how much do you like this slogan?"), which is reported in the main text, but also *brand attitude* ("Based on this slogan, what is your attitude toward the brand?"). In this robustness test, we replaced slogan liking with brand attitude. Despite these differences, the results were similar (see Table A8).

Table A8. Robustness test.

	Dependent Variable	
Predictor	(1) Brand Attitude	(2) Slogan Memory
Control: Brand Familiarity	0.26*** (0.01)	0.49* (0.19)
Linguistic Variable		
Slogan Length	-0.01 (0.01)	1.29*** (0.15)
Word Frequency	0.18* (0.08)	-7.45*** (1.32)
Distinctiveness	0.08 (0.08)	-1.58 (1.32)
Concreteness	-0.06 (0.05)	4.30*** (0.85)
Brand Name	-0.28*** (0.05)	7.26*** (0.93)
Intercept	2.85*** (0.41)	92.63*** (7.16)
R^2	0.46***	0.22***

Note. Unstandardized coefficients; standard errors appear in parentheses. Slogan Liking was rated on a scale from 1 (dislike) to 7 (like); Slogan Memory is % correct (Hits – False Alarms). * $p < .05$; *** $p < .001$.

The overall model remained highly significant, with comparably large effect sizes, in both brand attitude and slogan memory. Of the five key linguistic variables, four were again significant in the robustness test, and in the same directions as in the original analyses. The sole exception was distinctiveness, which was in the same direction as in the original analyses, but was no longer significant in predicting either brand attitude or slogan memory.

VIII. Online Replication and Combined Analysis

We tested the reliability of the results reported in the main text by conducting an exact replication with an online sample of 404 participants on Mechanical Turk. See Table A9 for results of this online replication, as well as results from the original lab sample and the online replication sample together. The results of the online replication were highly consistent with those of the main study; all five of the key linguistic variables significantly predicted liking and/or memory in this replication study. All five linguistic variables also predicted slogan liking and memory in the combined analysis.

Table A9. Regression results from the online replication of Study 1, and from a combined analysis of the original lab study (*N* = 594 students) and the online replication study (*N* = 404 Mechanical Turk workers).

	Online Replication		Combined Analysis	
Predictor	(1) Slogan Liking	(2) Slogan Memory	(3) Slogan Liking	(4) Slogan Memory
Control: Slogan Familiarity	0.39*** (0.02)	2.08*** (0.36)	0.44*** (0.02)	1.73*** (0.32)
Linguistic Variable				
Slogan Length	-0.02** (0.01)	1.61*** (0.19)	-0.01 (0.01)	1.49*** (0.15)
Word Frequency	0.12 (0.07)	-8.49*** (1.63)	0.18** (0.07)	-8.16*** (1.33)
Distinctiveness	0.16* (0.07)	-3.68* (1.64)	0.19** (0.07)	-2.61* (1.33)
Concreteness	-0.06 (0.04)	3.63*** (1.07)	-0.08† (0.04)	3.90*** (0.87)
Brand Name	-0.26*** (0.05)	9.30*** (1.16)	-0.31*** (0.05)	8.31*** (0.94)
Intercept	2.73*** (0.37)	82.36*** (8.94)	2.53*** (0.36)	87.45*** (7.25)
R^2	0.49***	0.22***	0.53***	0.26***

Note. Unstandardized coefficients; standard errors appear in parentheses. Slogan Liking was rated on a scale from 1 (dislike) to 7 (like); Slogan Memory is % correct (Hits – False Alarms). † $p < .075$, * $p < .05$, ** $p < .01$, *** $p < .001$.

IX. Interaction Model with Brand Familiarity

Brand familiarity might interact with the linguistic variables of interest in important ways. For instance, the linguistic effects of slogans might be accentuated among less familiar brands, or perhaps among more familiar brands with which consumers already have much experience. To test this possibility, we conducted a stepwise regression in which we entered our linguistic model but with brand familiarity instead of slogan familiarity (i.e., brand familiarity, slogan length, word frequency, word distinctiveness, concreteness, and brand name) in the first block, and we entered in a second block five interaction terms corresponding to the interaction of brand familiarity with the each of the five linguistic properties.

Results revealed that the addition of the five interaction terms did not significantly improve the model fit for slogan liking, $\Delta R^2 = 0.005$, $F(5, 808) = 1.38$, $p = .23$. For slogan memory, however, the interaction model significantly outperformed the reduced model, $\Delta R^2 = 0.020$, $F(5, 808) = 4.18$, $p < .001$. Of the five interaction terms, only the brand familiarity × brand name interaction was significant, $b = 1.53$, SE = 0.42, $t = 3.63$, $p < .001$. The positive coefficient indicates that including the brand name in the slogan improves memory of the slogan more for highly familiar brands than for less familiar brands.

To explore this interaction further, we conducted bootstrap analyses (Hayes 2013, model 1, 10K samples) with brand name (0 = absent, 1 = present) as independent variable, memory accuracy as dependent variable, and brand familiarity as moderator. The interaction of brand familiarity and brand name was again significant, $b = 1.65$, SE = 0.45, $t = 3.70$, $p < .001$, replicating the result of the linear regression reported above. We then examined the interaction via floodlight analysis, shown in Figure A1. The effect of brand name on memory accuracy was significant at all levels of brand familiarity. Importantly, however, the more familiar the brand, the larger its effect on slogan memory. At the extremes, including a completely unfamiliar brand name in the slogan increased memory for that slogan by about 4%, whereas including a completely familiar brand name increased slogan memory by about 14%.

In sum, although brand familiarity did not moderate the linguistic effects on slogan liking, brand familiarity did moderate the effect of including the brand name on memory for the slogan.

Figure A1. Interaction of brand familiarity and brand name inclusion on memory for slogans. The solid line indicates the effect size and the dotted lines indicate CI_{95}.

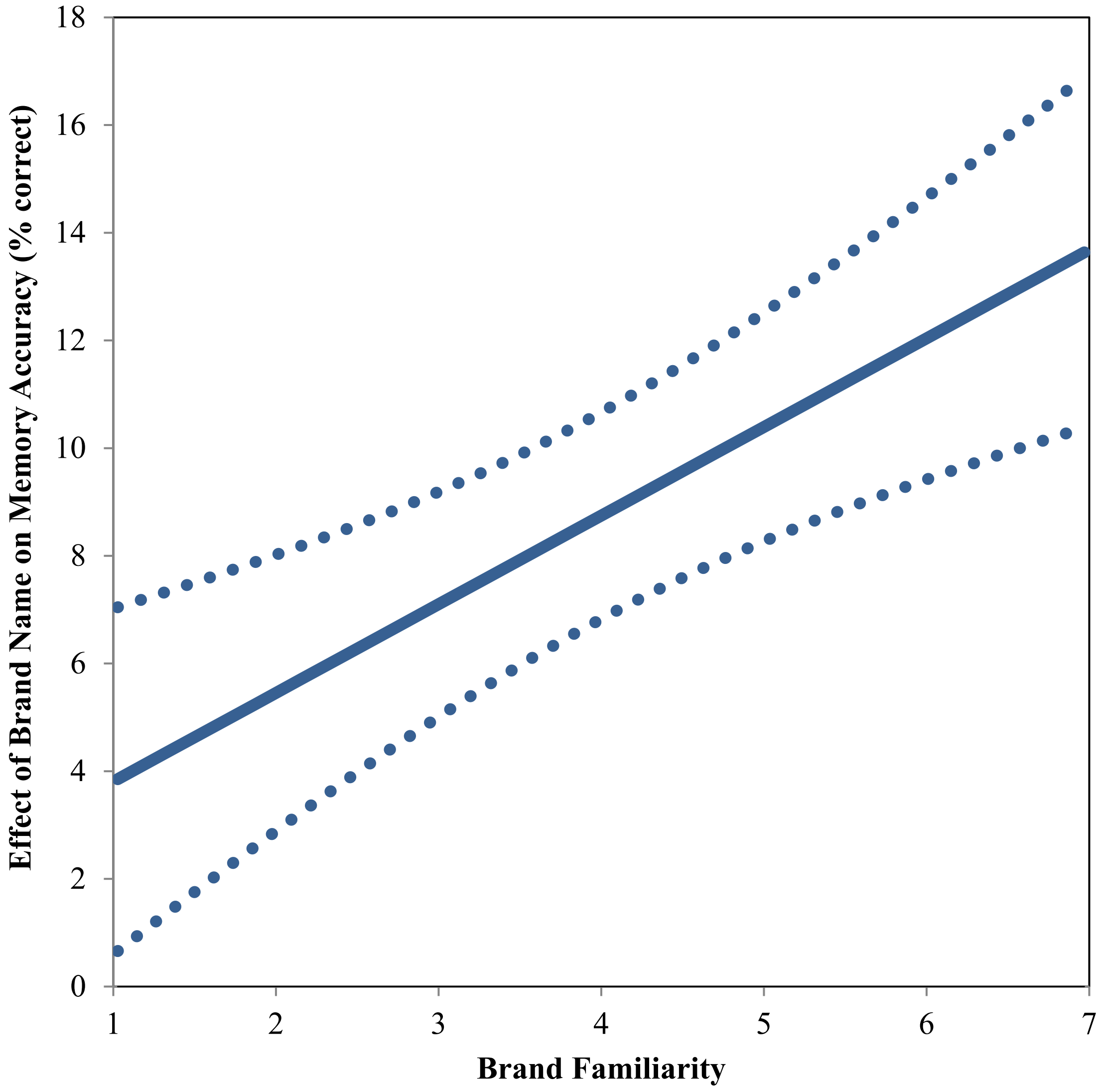

Web Appendix C: Slogan Stimuli in Studies 2-4

Study 2 Slogan Stimuli

Attitude Targets: Edited to Improve Slogan Attitudes

Brand	Slogan
Club Med	The antidote for civilization<br>*The cure for mankind
Budweiser	The genuine article<br>*The real deal
Western Airlines	The only way to fly<br>*The exclusive way to travel
Listerine	Stops halitosis<br>*Kills bad breath
Chevrolet	The road isn't built that can make it breathe hard<br>*No road can challenge it
Jacob's Club	If you like a lot of chocolate on your biscuit join our club<br>*Chocolate lovers wanted
Buick	When better automobiles are built, Buick will build them<br>*Building better automobiles
Fairy Dish Soap	Now hands that do dishes can feel soft as your face<br>*Clean dishes. Soft hands.
	Original<br>*Edited

Memory Targets: Edited to Improve Slogan Recognition

Brand	Slogan
Epiphany	See your way forward<br>*Envision your path forward
Toyota	Get the feeling<br>*Snag the sensation
Compaq	Has it changed your life yet?<br>*Has it transformed your world yet?
Butterfinger	Break out of the ordinary<br>*Flee the mundane
Hyundai	Always there for you<br>*Absolutely positively dependable
DHL	We keep your promises<br>*Your word is our wedding ring
Burger King	It just tastes better<br>*Simply souped up taste
Fancy Feast	Good taste is easy to recognize<br>*Your taste buds know the difference
	Original<br>*Edited

Brand Name Targets: Edited to Remove or Add the Brand Name

	Brand	Slogan
Brand Name Removed	Morrisons	More reasons to shop at Morrisons<br>*More reasons to shop
	Ice Lolly	What could be nicer than a Pendleton's Twicer<br>*What could be nicer?
	Watney's	What we want is Watney's<br>*What we want
	Anadin	Nothing is more effective than Anadin<br>*Nothing is more effective
	Horlicks	Horlicks guards against night starvation<br>*Guards against night starvation
		Original<br>*Edited
Brand Name Added	Hai Karate Aftershave	Be careful how you use it!<br>*Be careful how you use Hal Karate!
	Alkaseltzer	Try it, you'll like it<br>*Try Alkaseltzer, you'll like it
	Greggs	Ready when you are<br>*Greggs is ready when you are
	Cerebos	See how it runs!<br>*See how Cerebos runs!
		Original<br>*Edited

Study 3 Slogan Stimuli

Version*	Slogan
L	For a living planet (WWF)
M	For a living world
L	It's good to chat
M	It's good to talk (BT)
L	Echo around the globe
M	Echo around the world (Edison Records)
L	A vital part of your world (Tyco)
M	A crucial part of your world
L	The remedy for civilization
M	The antidote for civilization (ClubMed)
L	Don't you just love being in charge
M	Don't you just love being in control (British Gas)
L	I'd rather fight than switch (Tareyton)
M	I'd rather fight than change
L	The first time is never the tops
M	The first time is never the best (Campari)
L	Horlikcs guards against night starvation (Horlicks)
M	Horlicks guards against evening starvation
L	Now they whisper to her…not about her (Cashmere)
M	Now they gossip to her…not about her

*L=Less-fluent, M=More-fluent

Study 4 Slogan Stimuli

Version*	Slogan
L	The Genuine Article (Budweiser)
M	The Real Deal
L	The antidote for civilization (ClubMed)
M	The remedy for mankind
L	Snag the Sensation
M	Get the Feeling (Toyota)
L	Flee the Mundane
M	Break out of the Ordinary (Butterfinger)
B	Nothing is more effective than Anadin (Anadin)
N	Nothing is more effective
B	Try Alkaseltzer, you'll like it
N	Try it, you'll like it (Alkaseltzer)

*L=Less-fluent, M=More-fluent, B=Brand Name Included, N=No Brand Name

Study 4 Bumper Ads

The Genuine Article — Budweiser

The Real Deal — Budweiser

The antidote for civilization — Club Med

The remedy for mankind — Club Med

Get the Feeling — TOYOTA

Snag the Sensation — TOYOTA

Break out of the ordinary — Nestlé Butterfinger

Flee the Mundane — Nestlé Butterfinger

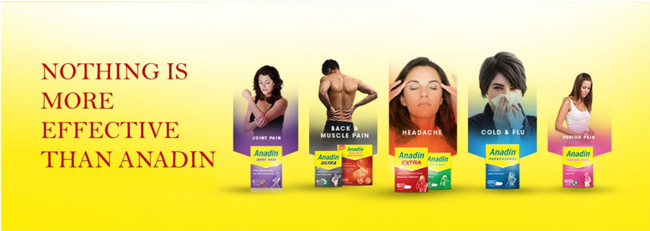

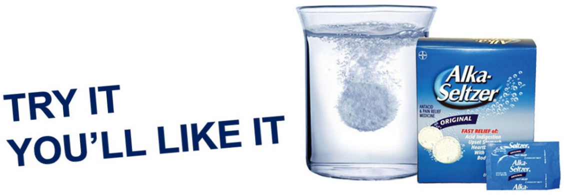

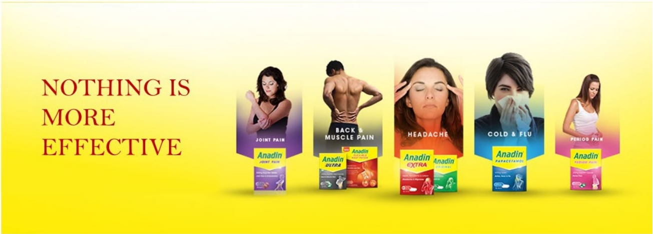

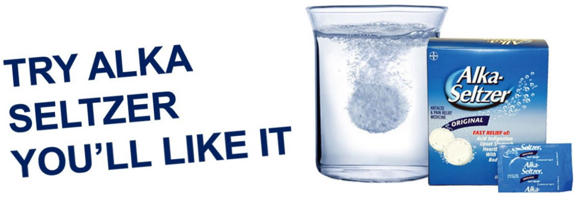

Web Appendix D: Linguistic Fluency Validation Measure

To validate our slogan edits, we created a measure that we called *linguistic fluency*, which is the extent to which a given slogan has linguistic properties associated with processing fluency. Based on the results of Study 1, we operationalized linguistic fluency as the extent to which the slogan contained relatively (a) few words, (b) frequent words, (c) distinctive words, and (d) abstract words. These are the linguistic properties shown in Study 1 to predict higher liking and lower memory. In Studies 2-5, we manipulated those properties to test whether they causally affect slogan liking and memory. This measure of linguistic fluency serves as a manipulation check of our slogan manipulations.

The four linguistic properties used different scales. For instance, slogan length is unbounded, theoretically ranging from one word to infinity, whereas concreteness was rated on a scale from 1-7 (Brysbaert et al. 2014). Thus, to create a single score of linguistic fluency that incorporated all four measures, we first *Z*-transformed all four measures.

In Study 1, some of the measures had larger effects (i.e., regression coefficients) than others. For instance, word frequency generally had larger effects on liking and memory than distinctiveness did. Because it was impossible to manipulate one property of a slogan without also affecting its other properties – e.g., manipulating frequency naturally affects distinctiveness, because they are moderately correlated – we sought to weight our measure of linguistic fluency such that the properties that had larger effects in Study 1 would carry more weight in our linguistic fluency manipulations of the slogans in Studies 2-5. Moreover, because we intended to affect both liking and memory, we wanted these factor weights to reflect the factors' influence on both liking and memory in Study 1.

Thus, to create a single weight (i.e., accounting for both liking and memory) for each the four variables, we averaged each factor's effects across liking and memory. We took the standardized coefficients (β) from the combined analysis of Study 1, reversed the sign of the coefficients for memory (so that higher numbers indicated higher fluency), and then simply averaged the two coefficients. To illustrate, distinctiveness had $\beta = .09$ in liking and $\beta = -.07$ in memory. If we simply averaged the two coefficients ($\beta_{average} = .01$), that would incorrectly imply that distinctiveness failed to predict liking and memory, when in fact it significantly predicted both liking and memory. By reversing the sign of the memory coefficient, however, we see that distinctiveness had a small but real combined effect on liking and memory ($\beta_{average} = .08$). Slogan length had a larger combined effect ($\beta_{average} = -.19$), whereas word frequency ($\beta_{average} = .165$) and concreteness ($\beta_{average} = -.095$) had moderate combined effects in opposite directions. Positive signs indicate that frequency and distinctiveness increase fluency, whereas negative signs indicate that slogan length and word concreteness decrease fluency.

Finally, for each slogan, we weighted each of the four linguistic variables (*Z*-transformed for common scale) by those averaged regression coefficients, and then summed the four weighted scores to produce a single measure of linguistic fluency that reflected its expected combined effect on liking and memory. This formula is expressed as follows:

$$\text{Linguistic Fluency} = \alpha\ \text{Length} + \beta\ \text{Frequency} + \gamma\ \text{Distinctiveness} + \delta\ \text{Concreteness}$$

where α, β, γ, and δ are the weights determined from the results of Study 1 (i.e., -.19, .165, .08, and -.095 respectively).

For example, Epiphany's slogan "See your way forward" had a length of 4 words, frequency of 3.78, distinctiveness of 1.51, and concreteness of 2.65, yielding a linguistic fluency score of .106:

“See your way forward” = (4 × -.19) + (3.78 × .165) + (1.51 × .08) + (2.65 × -.095) = .106

In Study 2, this slogan was one of our “memory targets;” i.e., we intended to decrease its linguistic fluency in order to increase memory of it. We therefore edited it to create an alternative slogan that was semantically similar but lower in linguistic fluency. For this particular slogan, we targeted word frequency as the primary variable to manipulate, with a decrease in frequency expected to decrease liking and increase memory. Our edited version of the slogan was as follows:

“Envision your path forward” = (4 × -.19) + (2.92 × .165) + (2.00 × .08) + (2.96 × -.095) = -.020

We used a similar procedure to edit the other slogans, although we targeted different variations of the four linguistic properties for each slogan. Across the sixteen pairs of attitude targets and memory targets in Study 2, the slogans that we intended to be more fluent indeed were significantly higher in linguistic fluency (M = .08, SD = .14) than the slogans that we intended to be disfluent (M = -.08, SD = .19), $t(30) = 2.72$, $p = .01$. Across the ten slogan pairs in Study 3, the slogans that we intended to be more fluent also were significantly higher in linguistic fluency (M = .11, SD = .14) than the slogans that we intended to be disfluent (M = -.11, SD = .13), $t(18) = 3.72$, $p < .01$. And, despite the small N, across the four slogan pairs in Study 4, again the slogans that we intended to be more fluent were indeed significantly higher in linguistic fluency (M = .13, SD = .18) than the slogans that we intended to be disfluent (M = -.13, SD = .08), $t(6) = 2.64$, $p < .05$. Also in Study 5, the slogan intended to be more fluent was indeed higher in linguistic fluency (M = .13) than the disfluent slogan (M = -.13). Thus, we successfully manipulated the linguistic fluency of the slogans in Studies 2-5.

Web Appendix E: Memory and Liking of Individual Slogans

To provide additional clarity on the relationship between memory and liking, we conducted contrasts on memory and liking for each individual slogan pair in addition to reporting the overall test statistics for these experimental stimuli groups in the manuscript.

Study 2: Attitude Targets

		Liking			Memory		
Brand	Slogan	Mean (SE)	F	*p*	Mean (SE)	F	*p*
Club Med	The antidote for civilization<br>*The cure for mankind	3.09 (.17)<br>3.69 (.17)	6.00	.02	.82 (.04)<br>.79 (.04)	.51	.47
Budweiser	The genuine article<br>*The real deal	3.43 (.15)<br>4.78 (.15)	37.82	.001	.69 (.04)<br>.64 (.04)	.66	.42
Western Airlines	The only way to fly<br>*The exclusive way to travel	4.22 (.15)<br>4.42 (.15)	.92	.34	.90 (.03)<br>.86 (.03)	.84	.36
Listerine	Stops halitosis<br>*Kills bad breath	2.56 (.15)<br>5.08 (.15)	148.4	.001	.95 (.02)<br>.93 (.02)	.72	.40
Chevrolet	The road isn't built that can make it breathe hard<br>*No road can challenge it	2.98 (.17)<br>4.72 (.18)	49.26	.001	.87 (.03)<br>.88 (.03)	.10	.75
Jacob's Club	If you like a lot of chocolate on your biscuit join our club<br>*Chocolate lovers wanted	2.86 (.18)<br>4.37 (.17)	36.89	.001	.94 (.03)<br>.88 (.03)	3.00	.08
Buick	When better automobiles are built, Buick will build them<br>*Building better automobiles	3.49 (.16)<br>4.26 (.16)	11.88	.001	.95 (.02)<br>.88 (.02)	3.73	.05
Fairy Dish Soap	Now hands that do dishes can feel soft as your face<br>*Clean dishes. Soft hands.	3.16 (1.9)<br>4.56 (1.9)	33.36	.001	.97 (.02)<br>.94 (.02)	1.29	.26
	Original<br>*Edited	3.22 (.11)<br>4.48 (.11)	230.1	.001	.89 (.03)<br>.85 (.03)	6.38	.01

Study 2: Memory Targets

Brand	Slogan	Liking Mean (SE)	Liking F	Liking *p*	Memory Mean (SE)	Memory F	Memory *p*
Epiphany	See your way forward<br>*Envision your path forward	4.17 (.16)<br>4.33 (.16)	.55	.46	.60 (.04)<br>.67 (.04)	1.47	.23
Toyota	Get the feeling<br>*Snag the sensation	3.96 (.17)<br>3.61 (.17)	2.20	.14	.41 (.04)<br>.75 (.04)	31.44	.001
Compaq	Has it changed your life yet?<br>*Has it transformed your world yet?	3.38 (.17)<br>3.92 (.17)	5.14	.02	.72 (.04)<br>.66 (.04)	.95	.33
Butterfinger	Break out of the ordinary<br>*Flee the mundane	4.92 (.16)<br>3.90 (.16)	19.32	.001	.51 (.04)<br>.83 (.04)	31.85	.001
Hyundai	Always there for you<br>*Absolutely positively dependable	4.30 (.16)<br>4.14 (.16)	.53	.47	.58 (.04)<br>.84 (.04)	20.58	.001
DHL	We keep your promises<br>*Your word is our wedding ring	4.36 (.17)<br>3.06 (.17)	28.06	.001	.74 (.03)<br>.93 (.03)	17.60	.001
Burger King	It just tastes better<br>*Simply souped up taste	3.80 (.16)<br>2.59 (.15)	30.40	.001	.82 (.03)<br>.93 (.03)	5.91	.02
Fancy Feast	Good taste is easy to recognize<br>*Your taste buds know the difference	4.23 (.16)<br>4.40 (.16)	.56	.46	.59 (.04)<br>.89 (.04)	31.53	.001
	Original<br>*Edited	4.14 (.15)<br>3.74 (.15)	22.54	.001	.62 (.04)<br>.81 (.04)	94.43	.001

Study 2: Brand Name Targets

	Brand	Slogan	Liking Mean (SE)	Liking F	Liking p	Memory Mean (SE)	Memory F	Memory p
Brand Name Removed	Morrisons	More reasons to shop at Morrisons	3.20 (.16)	4.54	.03	.95 (.03)	48.45	.001
		*More reasons to shop	3.67 (.16)			.61 (.03)		
	Ice Lolly	What could be nicer than a Pendleton's Twicer	2.75 (.16)	4.27	.04	.80 (.04)	14.82	.001
		*What could be nicer?	3.22 (.16)			.58 (.04)		
	Watney's	What we want is Watney's	2.88 (.16)	.17	.68	.91 (.04)	77.06	.001
		*What we want	2.98 (.16)			.45 (.04)		
	Anadin	Nothing is more effective than Anadin	2.57 (.14)	4.59	.03	.89 (.04)	32.29	.001
		*Nothing is more effective	3.01 (.15)			.58 (.04)		
	Horlicks	Horlicks guards against night starvation	2.88 (.17)	7.27	.01	.91 (.03)	.32	.57
		*Guards against night starvation	3.52 (.17)			.89 (.03)		
		Original	2.85 (.12)	17.99	.001	.89 (.04)	137.3	.001
		*Edited	3.28 (.12)			.62 (.04)		
Brand Name Added	Hai Karate	Be careful how you use it!	3.13 (.17)	2.17	.14	.70 (.03)	29.16	.001
		*Be careful how you use Hal Karate!	2.77 (.18)			.95 (.03)		
	Alkaseltzer	Try it, you'll like it	3.70 (.16)	13.19	.001	.81 (.03)	10.55	.001
		*Try Alkaseltzer, you'll like it	2.90 (.15)			.94 (.03)		
	Greggs	Ready when you are	4.13 (.17)	14.03	.001	.63 (.04)	28.56	.001
		*Greggs is ready when you are	3.22 (.17)			.91 (.04)		
	Cerebos	See how it runs!	2.64 (.14)	.64	.42	.47 (.04)	51.33	.001
		*See how Cerebos runs!	2.48 (.14)			.86 (.04)		
		Original	3.40 (.25)	23.74	.001	.65 (.05)	115.1	.001
		*Edited	2.84 (.25)			.92 (.05)		

Study 4a and 4b: YouTube Banner Ads Slogan Stimuli

		Liking			Memory		
Brand	Slogan	Mean (SE)	F	*p*	Mean (SE)	F	*p*
Budweiser	The genuine article<br>*The real deal	3.36 (.12)<br>4.62 (.11)	58.86	.001	.42 (.03)<br>.52 (.03)	4.89	.03
ClubMed	The antidote for civilization<br>*The remedy for mankind	3.12 (.12)<br>3.72 (.13)	11.37	.001	.44 (.03)<br>.44 (.03)	.01	.93
Toyota	Snag the sensation<br>*Get the feeling	3.37 (.13)<br>3.50 (.13)	.52	.47	.53 (.03)<br>.35 (.03)	16.67	.001
Butterfinger	Flee the mundane<br>*Break out of the ordinary	3.25 (.13)<br>4.72 (.13)	63.68	.001	.67 (.03)<br>.50 (.03)	16.51	.001
Anadin	Nothing is more effective than Anadin<br>*Nothing is more effective	3.30 (.13)<br>3.81 (.12)	8.57	.004	.68 (.03)<br>.29 (.03)	89.39	.001
Alkaseltzer	Try Alkaseltzer, you’ll like it<br>*Try it, you’ll like it	2.92 (.14)<br>3.52 (.14)	9.18	.003	.81 (.03)<br>.55 (.03)	39.33	.001
	Less-fluent<br>*More-fluent	3.22 (.13)<br>3.98 (.13)	105.2	.001	.59 (.04)<br>.44 (.04)	71.65	.001

Web Appendix F: Study 2 Replication—Brand Name Repetition

As an additional robustness test of the brand name added/removed slogan condition in Study 2, we conducted a replication of Study 2 in which we removed the brand name from being shown beneath all of the slogans. It was brought to our attention that a potential alternative explanation for the increased memory we find for slogans that included the brand name (e.g., Try Alka-Seltzer, you'll like it) is that individuals see the brand name twice in these slogans (i.e., embedded in the slogan and paired with the slogan). While this is often the case in real life, it nonetheless warrants further investigation in order to theoretically rule out that our effects for the brand name slogans in Study 2 were not merely driven by this repetition of the brand name.

Method

Participants. Five hundred twenty-nine undergraduates at a U.S. university participated.

Stimuli. We used the same 25 slogan pairs as in Study 2, but added an additional between-subjects condition in which the brand name no longer appeared beneath the slogans for half of the participants.

Procedure. The procedure was the same as in Study 2, except that half the participants (i.e., those who were not shown the brand name beneath the slogan) did not rate brand familiarity.

Results

The results of Study 2 were replicated. Specifically, when participants saw the slogans with the brand names paired beneath the slogans (i.e., a repeat of our Study 2 design) they again demonstrated increased memory for slogans that contained the brand name (*Brand Name Removed Targets*: $M_{\text{Original}} = .84$, SE = .03, $M_{\text{Edited}} = .59$, SE = .03, $b = .24$, $t(1318.69) = 10.23$, $p < .001$. *Brand Name Added Targets*: $M_{\text{Edited}} = .87$, SE = .06, $M_{\text{Original}} = .64$, SE = .06, $b = .23$, $t(1054.98) = 9.26$, $p < .001$). When participants saw the slogans without the brand name paired beneath the slogans, the results again replicated (*Brand Name Removed Targets:* $M_{\text{Original}} = .90$, SE = .02, $M_{\text{Edited}} = .77$, SE = .02, $b = .13$, $t(1313.83) = 6.36$, $p < .001$. *Brand Name Added Targets*: $M_{\text{Edited}} = .93$, SE = .03, $M_{\text{Original}} = .81$, SE = .03, $b = .13$ $t(1050.86) = 6.37$, $p < .001$). In sum, regardless of whether the brand name was shown beneath the slogan or not, participants better remembered slogans that embedded the brand name in the slogan. Repetition of the brand name, therefore, cannot explain the effects on memory in Study 2.

We also note that the results of the other stimuli (e.g., "attitude targets", "memory targets"), as well as the results of the slogan liking dependent variable, also replicated the pattern reported in the main Study 2. Results of the condition in which the brand name did *not* appear beneath each slogan are shown in Figure A2, demonstrating the same pattern of significant results as in the main Study 2 (where the brand name *did* appear beneath each slogan). In sum, removing the brand name from beneath the slogan had no effect on the pattern of significant effects on either liking or memory of the slogans.

Figure A2. (**A**) Mean attitude ratings and (**B**) memory accuracy (proportion correct). Study 2 replication (no brand name). Error bars indicate ±1 *SE*.

A.

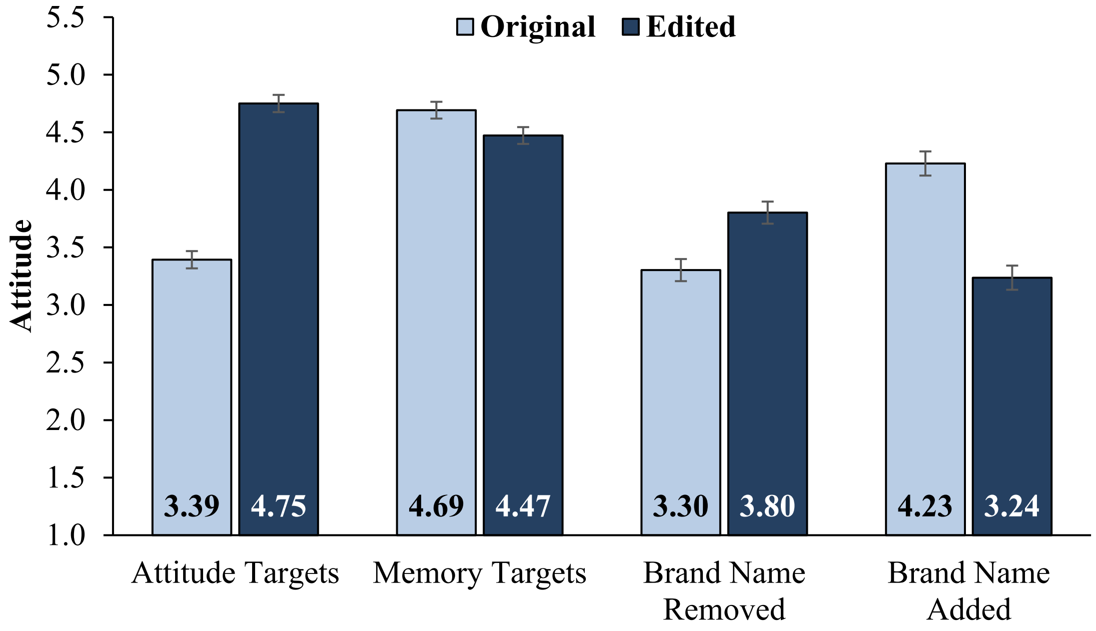

B.

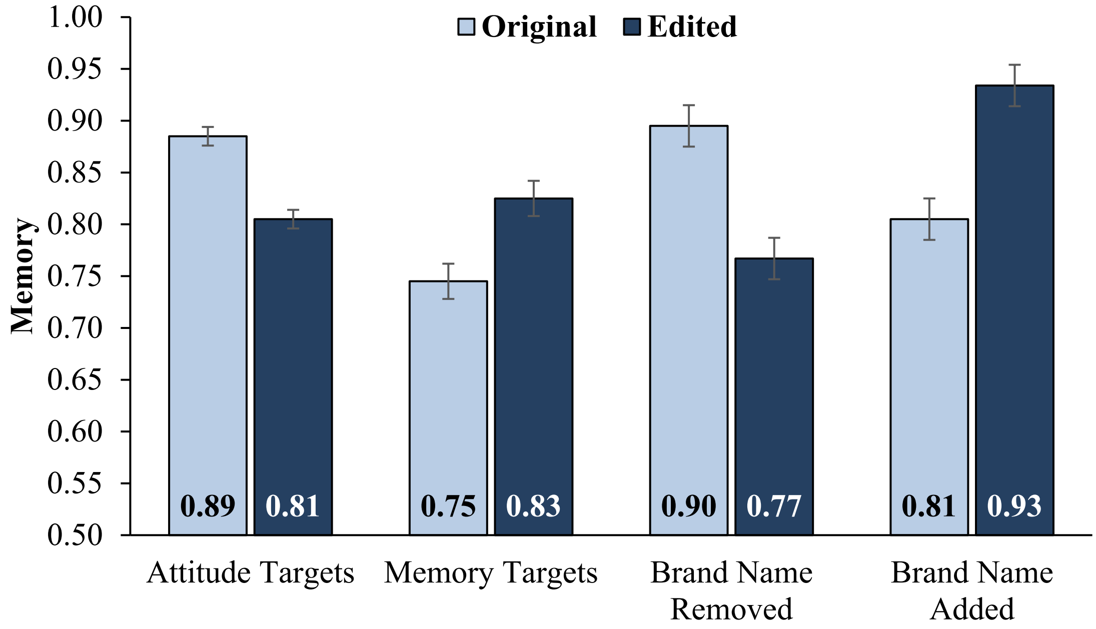

Web Appendix G: Study 4b Replication—Slogan Recall

As an additional robustness test of our Study 4 results, we conducted a modified version of Study 4b that employed an alternative memory measure—free recall. Specifically, we were interested in how often the manipulated target words were correctly recalled in the disfluent vs. fluent versions of the slogans after incidental exposure during the brief YouTube bumper ads.

Method

Participants. Five hundred thirty-two undergraduates (44% females) at a U.S. university participated.

Stimuli. We used the same six bumper ads as in Study 4b, minus the two brand-name added/removed bumper ads (the latter differed only on the presence or absence of a single brand word, precluding them from target word recall comparison). We also decreased the number of filler ads to a single filler ad (the first filler ad in Study 4b) to reduce cognitive load.

Procedure. We altered the surprise memory test in the final step of the experiment to be a free recall task instead of the previous recognition task. All else was identical to Study 4b.

For the surprise recall task, we presented participants with a scrubbed version of each bumper ad with the slogan removed and asked them to "please write the slogan that you remember seeing in this advertisement (the slogan has been removed)". Participants responded to each advertisement one by one in the same order as they were presented during the YouTube video portion of the study.

Results

Two graduate research assistants at a U.S. university independently coded the free recall responses for the number of target words correctly recalled in each bumper ad slogan. Because each slogan contained two manipulated target words, participants' responses were coded as 0, 1, or 2 (2=correctly recalled both target words) for each slogan. Memory accuracy was then calculated as the proportion of correctly recalled slogan words within the fluent and disfluent conditions separately.

The results fully corroborated those of Study 4b, with participants better recalling the disfluent versions ($M = .17$, $SE = .01$) of the slogans than the fluent versions ($M = .12$, $SE = .01$), $t(531) = 4.43$, $p < .001$, and confirming once again that slogans with less-fluent words are better remembered, even when individuals are incidentally exposed to the slogans in a more realistic context.
